# Zirconium-Based Metal–Organic Frameworks for Photocatalytic CO_2_ Reduction

**DOI:** 10.1021/prechem.5c00009

**Published:** 2025-05-06

**Authors:** Mei Li, Hao Zhang, Cha Li, Feifan Lang, Shi-Wei Yao, Jiandong Pang, Xian-He Bu

**Affiliations:** † School of Materials Science and Engineering, Smart Sensing Interdisciplinary Science Center, Tianjin Key Laboratory of Metal and Molecule-Based Material Chemistry, Collaborative Innovation Center of Chemical Science and Engineering, 12538 Nankai University, Tianjin 300350, China; ‡ State Key Laboratory of Elemento-Organic Chemistry, Frontiers Science Center for New Organic Matter, College of Chemistry, Nankai University, Tianjin 300071, China; # Haihe Laboratory of Sustainable Chemical Transformations, Tianjin 300192, China; § State Key Laboratory of Heavy Oil Processing, School of Materials Science and Engineering, 74591 China University of Petroleum (East China), Qingdao 266580, China

**Keywords:** CO_2_ photoreduction, Zr-MOFs, catalytic mechanisms, regulation strategies, advanced characterizations

## Abstract

Photocatalytic carbon dioxide (CO_2_) reduction shows great potential as an important approach to tackling global energy and environmental challenges. In recent years, zirconium-based metal–organic frameworks (Zr-MOFs), as an emerging class of crystalline porous solid materials, have attracted much attention in the field of photocatalytic CO_2_ reduction due to their unique tailorable structures, high surface areas, and exceptional stability. In this Review, we first provide an in-depth discussion on the semiconductor-like behavior of Zr-MOFs and their fundamental mechanisms in photocatalytic CO_2_ reduction. Subsequently, we systematically summarize current frontier strategies for enhancing the photocatalytic activity of Zr-MOFs, which include but are not limited to improving light absorption and utilization efficiency, promoting effective separation and transportation of photogenerated charges, and optimizing the surface redox reaction process. Furthermore, we elaborate on some advanced characterization techniques that can precisely track reaction intermediates and profoundly reveal the photocatalytic reaction kinetics within the Zr-MOF system. Finally, we propose possible future challenges and potential research directions for the development of Zr-MOFs in photocatalytic CO_2_ reduction, aiming to provide valuable insights for researchers in related fields.

## Graphical abstract

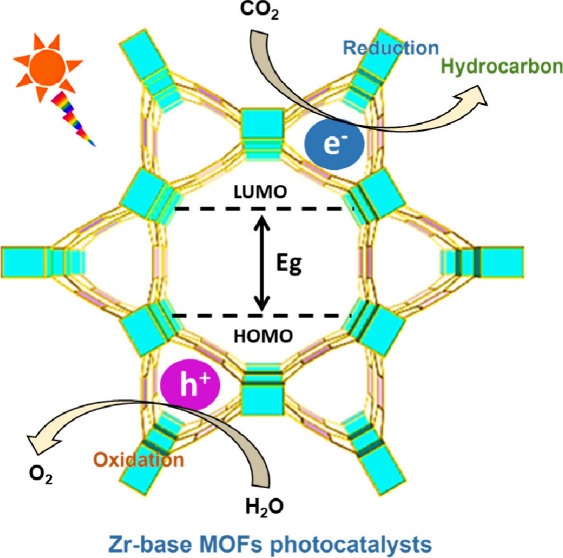

## 1 Introduction

With rapid economic development and improvement in living standards, the total energy consumption in human society has continued to increase. Fossil fuels remain the primary component of the human energy supply to date. However, as nonrenewable resources, fossil fuels are being depleted at an accelerating rate due to human consumption, which poses a global energy crisis. Concurrently, their use also results in pollution and the emission of CO_2_ greenhouse gases.^1–5^ To meet the growing global energy demand, there is an urgent need to develop renewable clean energy sources that can mitigate both energy scarcity and environmental pollution.^6–12^ Solar energy, characterized by its renewability, abundance, efficiency, and environmental friendliness, has been widely recognized as one of the most promising technologies and strategies to address these challenges by converting clean energy from sunlight.^13–19^

In natural photosynthesis, green plants utilize light energy to convert CO_2_ and H_2_O into energetic organic compounds while releasing oxygen. Inspired by this process, artificial photosynthesis has been proposed and presents a promising approach for converting CO_2_ into valuable fuels and chemical precursors, such as CO, CH_3_OH and HCOOH.^20–25^ Artificial photosynthesis primarily consists of three fundamental processes. First, the generation of electron–hole pairs, which occurs when the photon energy absorbed by the photocatalyst exceeds its bandgap energy, prompting electrons to transition from the valence band to the conduction band and leaving behind holes in the valence band, thereby forming electron–hole pairs. Second, the efficient separation and transfer of electron–hole pairs to reactive sites on the catalyst surface. Third, the isolated photogenerated electron–hole pairs engage individually in redox reactions. Photocatalysts, both semiconductor and semiconductor-like materials, constitute the cornerstone of artificial photosynthesis. A series of semiconductors, such as C_3_N_4_, TiO_2_, CdS, Bi_2_WO_6_, and their composites have been developed and demonstrated high photocatalytic performance and stability.^26–30^ However, most semiconductors suffer from insufficient light harvesting due to the wide bandgap, rapid recombination of electron hole pairs, and limited exposure of active sites (Figure 1a).

**Figure 1:**
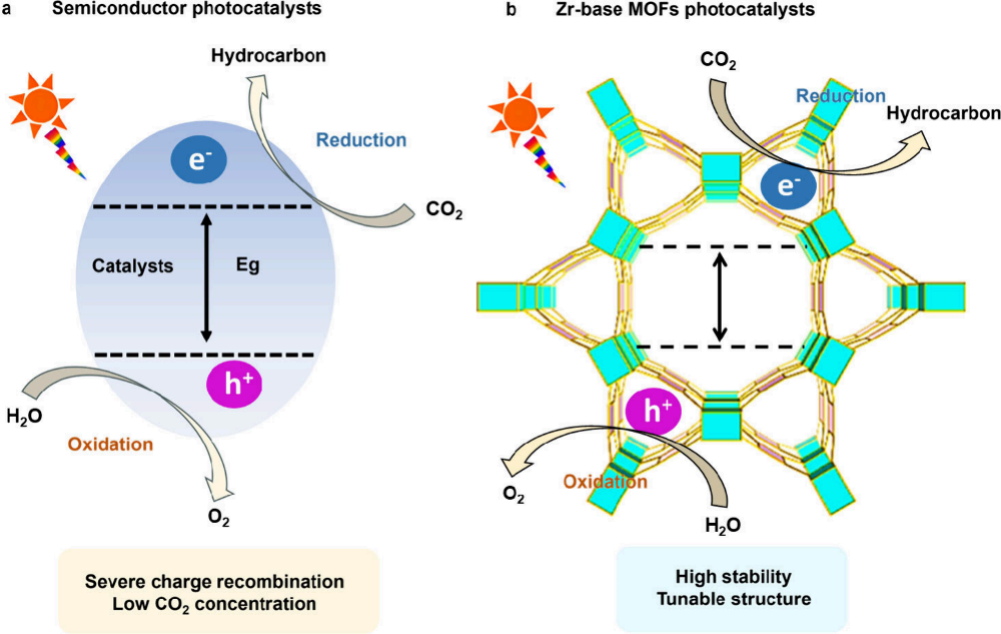
Artificial photosynthesis reaction system of (a) a semiconductor and (b) Zr-based MOFs.

Recently, great efforts have been devoted to developing innovative and efficient catalysts, encompassing metal-doped zeolites,^31,32^ transition metal complexes,^33,34^ covalent–organic frameworks (COFs)^35,36^ and metal–organic frameworks (MOFs).^37,38^ The easily adjustable and modified pore structure of MOF enhance the exposure of active sites and CO_2_ adsorption.^39–42^ Given the well-defined and tailorable pore structures, MOFs are assumed to serve as an ideal platform to understand how microenvironment modulation around catalytic sites affects the catalysis.^43,44^ Furthermore, the versatility offered by structural and functional ligands provides an ideal platform for bandgap engineering and the integration of catalytic centers.^45^ Meanwhile, the atomically precise structure of MOFs enables real-time monitoring of the catalytic reaction process and the capture of reaction intermediates, which contributes to the study of the catalytic mechanism.^46,47^ However, MOFs-based photocatalysts suffer from the problem of poor stability, which makes it difficult to maintain their porous structures during prolonged light exposure or in the presence of water. Moreover, the complex crystal and electronic structures of MOFs result in unclear charge transport pathways, further complicating their photocatalytic process. Zr-MOFs are of general interest due to their high thermal and chemical stability, which allows them to cope with complex and harsh catalytic conditions.^48–62^ Their semiconducting properties facilitate the selective catalytic reduction of CO_2_ through ligand-to-Zr_6_O_8_ cluster charge transfer (LMCT) or the catalytic activity of the ligands themselves (Figure 1b). However, the visible light response of Zr-MOFs is mainly dependent on the functional ligand due to the large bandgap of the Zr_6_ oxygen clusters. Unfortunately, Zr-MOFs are not effective utilization of solar energy due to the fact that the commonly available aromatic polycarboxylate ligands are only responsive to ultraviolet light (only 4% of the entire solar spectrum).^63,64^ And most Zr-MOFs exhibit low photocatalytic CO_2_ reduction efficiency due to poor charge carrier mobility resulting from their fast electron–hole pair recombination.^65,66^ Moreover, the difficulty in distinguishing and determining the roles of metal nodes, organic ligands, and their interfaces in photocatalysis of Zr-MOFs, as well as the limitations of the existing characterization techniques, make the study of the photocatalytic mechanism and the design of high-performance Zr-MOF photocatalysts challenging. Therefore, it is crucial to gain an in-depth understanding of the charge transfer and catalytic mechanism of Zr-MOF photocatalytic CO_2_ reduction by combining advanced characterization techniques, and to analyze and summarize the strategies to improve the photocatalytic performance.

The application of Zr-based MOFs for artificial photosynthetic CO_2_ reduction is still an emerging field and has attracted significant research attention. In this Review, we comprehensively summarize and discuss the recent progress in employing Zr-MOFs for photocatalytic CO_2_ reduction. Herein, we first elucidate the fundamental characteristics of Zr-MOFs as photocatalysts for CO_2_ reduction including their semiconductor-like behavior and catalytic mechanisms. The strategies to improve the photocatalytic performance are subsequently detailed. Additionally, the advanced characterization techniques for investigating photocatalytic kinetics, monitoring reaction intermediates, and assessing charge transfer processes are discussed. Finally, we propose the challenges and future prospects pertaining to the application of Zr-MOFs in photocatalytic CO_2_ reduction.

## 2 Mechanism Study of Zr-MOFs as Photocatalysts for CO_2_ Reduction

The photocatalytic performance of Zr-MOFs is significantly influenced by their optical absorption capabilities in both ultraviolet and visible light spectra, as well as their inherent band-edge energies.^67,68^ The photoluminescence investigations have elucidated that the Zr-metal clusters embedded within MOF frameworks can be considered as inorganic semiconductor quantum entities.^69,70^ These Zr metal nodes are photosensitized by two main mechanisms. One is the organic linkers act as photosensitizers, facilitating the absorption of light energy and subsequently triggering activation of these semiconductor quantum dots via ligand-to-metal charge transfer (LMCT) or metal-to-ligand charge transfer (MLCT) mechanism.^71,72^ The other is achieved using an external photosensitizer present within the reaction medium or anchored onto the Zr-MOFs. More importantly, the microporous and mesoporous structures inherent in MOFs play a crucial role by offering an exclusive platform for the adsorption and activation of substrates for subsequent redox reactions. As depicted in Figure 2, the photocatalytic process involving Zr-MOFs as catalysts typically proceeds through three essential stages:

**Figure 2:**
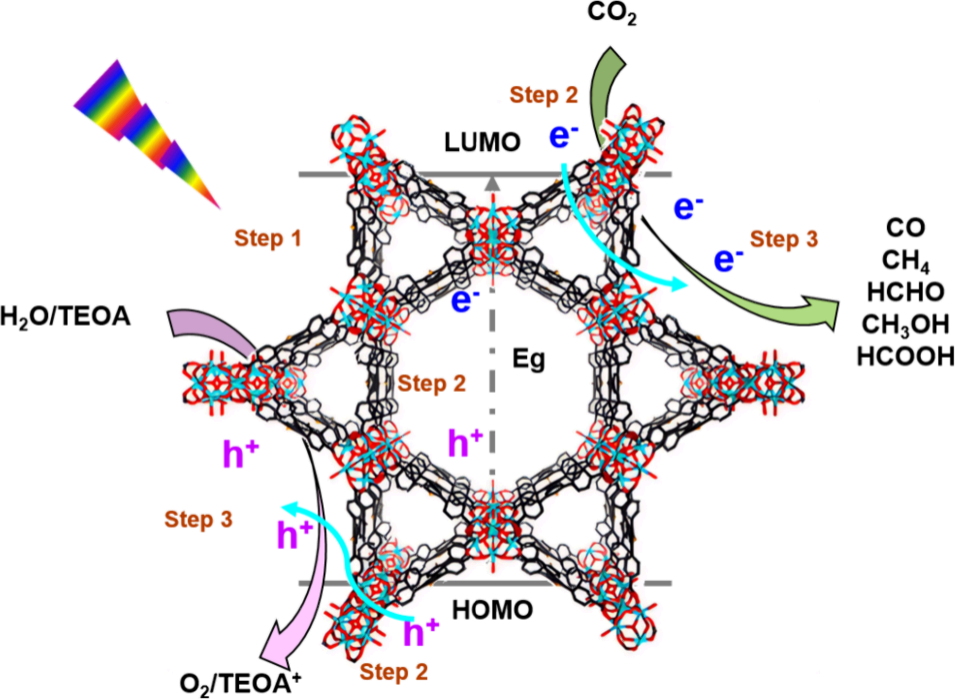
Zirconium-based metal–organic frameworks for photocatalytic CO_2_ reduction.

**(1) Light Absorption and Separation of Photoinduced Electron–Hole Pairs:** The catalyst first undergoes light adsorption process under light irradiation, then photogenerated electron–hole pairs are generated when the absorbed photon energy (*hv*) surpasses the bandgap energy of the Zr-MOFs, leading to the excitation of photogenerated electrons in the conduction band and the corresponding formation of holes in the valence band, thus electron–hole separation occurs.

**(2) Migration of Photogenerated Electrons and Holes:** The photogenerated electrons and holes migrate toward specific reaction sites through pathways such as LMCT and MLCT. For using additional photosensitizers or metal catalytic centers in Zr-MOFs, the photocatalytic activity is affected by the direct transfer of charge carriers to the catalytic center.

**(3) Redox Reactions:** At the specific reaction sites, oxidation or reduction half-reactions occur, matching the required redox equivalents.

The photocatalytic performance of Zr-MOFs is primarily influenced by three critical factors. First, the positions of the conduction band minimum (CBM) and valence band maximum (VBM), along with their respective redox potentials, determine the capability of photogenerated electron–hole pairs to participate in redox reactions.^73–75^ A more positive CBM or a more negative VBM enhances reduction or oxidation capabilities, respectively.^76,77^ These photoredox potentials directly correlate with the specific redox reactions targeted by the Zr-MOFs. Second, the efficiency of photocatalysis is profoundly affected by the lifetime of photogenerated charge carriers.^78,79^ The recombination of electrons-holes, occurring both within the bulk phase (bulk recombination) and on the surface (surface recombination) of Zr-MOFs, must be minimized to prolong carrier lifetimes and enhance charge transfer efficiency. Lastly, effective CO_2_ adsorption and activation are crucial for achieving superior photocatalytic performance.^80,81^ Based on the electron–hole separation efficiency and the redox capability of the conduction and valence bands, the general pathways for CO_2_ photoreduction are as follows:

1 $${\mathrm{CO}}_{2}+2H^{+}+2e^{-}\to \mathrm{CO}+H_{2}O,,E^{\theta }=-0.53\;V$$

2 $${\mathrm{CO}}_{2}+2H^{+}+2e^{-}\to \mathrm{HCOOH},,E^{\theta }=-0.61\;V$$

3 $${\mathrm{CO}}_{2}+4H^{+}+4e^{-}\to \mathrm{HCHO}+H_{2}O,,E^{\theta }=-0.48\;V$$

4 $${\mathrm{CO}}_{2}+6H^{+}+6e^{-}\to {\mathrm{CH}}_{3}\mathrm{OH}+H_{2}O,,E^{\theta }=-0.38\;V$$

5 $${\mathrm{CO}}_{2}+8H^{+}+8e^{-}\to {\mathrm{CH}}_{4}+2H_{2}O,,E^{\theta }=-0.24\;V$$

## 3 Strategies for Enhancing Photocatalytic Activity

The photocatalytic mechanism of Zr-MOFs can be simply described as the process of light capture, photoinduced electron–hole separation and transfer, and subsequent redox reactions. To enhance this process, various strategies are employed: such as bandgap engineering,^82,83^ morphology tailoring^84,85^ and modifying the design of Zr-MOF materials.^86,87^ The bandgap engineering strategy, a prevalent approach, is commonly achieved by incorporating functional moieties within the framework, such as photoactive components, additional metal sites, or secondary building units, to enhance light harvesting and charge separation in Zr-MOFs. Another effective approach focuses on designing MOF hybrid materials with suitable band alignments to promote photogenerated charges separation and match desired redox potential.^88,89^ The bandgap of the photocatalyst dictates the energy required for charge separation, while the positions of the conduction band (CB) and valence band (VB) crucially influence its oxidation and reduction capabilities. Enhancing carrier mobility facilitates effective separation and migration of photogenerated electrons and holes, thereby reducing recombination and prolonging their lifetimes This is essential for improved photocatalytic performance. Morphological engineering plays a pivotal role here, encompassing strategies such as tuning crystal shape or size, developing two-dimensional Zr-MOF nanosheet, and surface modification.^90,91^ In addition to light capture and carrier transfer, efficient adsorption and activation of CO_2_ on the Zr-MOF catalyst surfaces are critical for conversion efficiency. In the CO_2_ photoreduction process, a competing H_2_ evolution reaction occurs. Increasing CO_2_ concentration at reaction sites is crucial for enhancing activity and selectivity.^92,93^ Incorporating amino functional groups within Zr-MOF frameworks or interacting CO_2_ with O or C atoms in MOFs has proven effective in boosting CO_2_ adsorption and activation.^94,95^ These methodologies have demonstrated significant efficacy in enhancing photocatalytic performance and will be further elucidated in subsequent sections.

### 3.1 Enhanced Light Capture

Light absorption and photon-to-electron conversion are fundamental requirements for photocatalysis. Among various factors influencing photocatalytic performance, the effective regulation of visible light absorption capacity stands out as pivotal. The range of light absorption and light-capturing capacity can be broadened and improved by adjusting the bandgap of photocatalysts or introducing a second or more chromophore unit into the photocatalyst. A number of strategies have been exploited and discussed as below.

#### 3.1.1 Linker Functionalization

Integrating photosensitive components into pristine Zr-MOFs via covalent bonds or supramolecular interactions has proven an effective strategy for enhancing photocatalytic activity, especially when the intrinsic photoactive component is lacking in the pristine material.^96^ In recent advancements in MOFs chemistry, amino-functionalization has emerged as a prominent approach. For instance, Chen and his colleagues^97^ pioneered the construction of Zr-SDCA-NH_2_, a porous Zr-MOFs responsive to visible light, by using amine-functionalized dicarboxylic acid ligands and Zr clusters. Under visible light irradiation, Zr-SDCA-NH_2_ exhibited remarkable efficiency in converting CO_2_ to formic acid (Figure 3a). This photocatalytic reaction was achieved using triethanolamine (TEOA) as a sacrificial agent in a CH_3_CN solvent, with a HCOOH conversion rate as high as 96.2 μmol·h^–1^·mmol_MOF_^–1^, surpassing the rates reported for other amine-functionalized Zr-MOFs. The UV–vis diffuse reflectance spectroscopy (UV-DRS) confirmed that the introduction of amino groups enabled Zr-SDCA-NH_2_ to broaden its visible light absorption range, with an absorption edge around 600 nm and a narrowed band gap. This modification narrowed the band gap, significantly enhancing both the light absorption and electron transfer efficiency (Figure 3b,c). Hu and his colleagues^98^ proposed a simple one-pot solvothermal strategy for the preparation of amino-functionalized MOF (aU­(Zr/In)) (Figure 3d). The photoreduction of CO_2_ was evaluated under visible light irradiation (λ > 420 nm) in a gas–solid reaction system. As shown in Figure 3e, U­(Zr) was not active in the CO_2_ photoreduction. The photocatalytic activity was activated by amino modification and further promoted by In doping. A series of photophysical and photoelectrochemical characterizations demonstrated that amino modification and doping improved light absorption, charge separation, as well as interfacial charge transfer and prolonged the lifetime of photogenerated carriers (Figure 3f).

**Figure 3:**
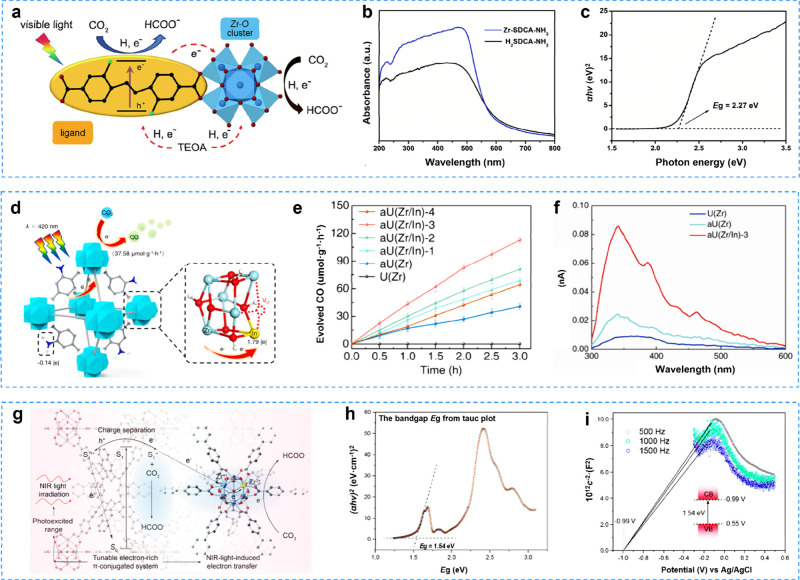
**a** Photocatalytic CO_2_ reduction for Zr-SDCA-NH_2_ under visible light irradiation; **b** UV–vis spectra of Zr-SDCA-NH_2_ and H_2_SDCA-NH_2_ ligand; **c** tauc plot of Zr-SDCA-NH_2_. Reproduced with permission from ref 97. Copyright 2018, Royal Society of Chemistry. **d** aU­(Zr/In) for CO_2_ photoreduction under NIR-; **e** time courses of CO evolution; and **f** SPC spectra. Reproduced with permission from ref 98. Copyright 2023, Elsevier. **g** TNP-MOF for CO_2_ photoreduction under NIR-light; **h** Tauc plot analysis showing the band gap of TNP-MOF; and **i** Mott–Schottky plot. Reproduced with permission from ref 101. Copyright 2022, American Chemical Society.

The photoactivation and ligand-to-cluster charge transfer processes of electron-rich conjugated linkers as well as the high CO_2_ adsorption capacity contribute to the improvement of the photocatalytic performance. Porphyrins, as classic electron-rich conjugated linkers, have attracted researchers developed a series of porphyrin-based metal–organic frameworks (PMOFs).^99^ These PMOFs extend the light absorption range into the visible spectrum through their excellent chromophoric properties. As an illustrative example, a porphyrin-involved PCN-222 MOFs exhibited remarkable photocatalytic CO_2_ conversion to HCOOH, attributed primarily to its exceptional CO_2_ capture capacity, broad range of light absorption, and efficient electron–hole separation.^100^ In 2022, Zhang’s group^101^ constructed a near-infrared (NIR) light-driven photocatalyst, TNP-MOF, by using a large π-conjugated porphyrin ligand and Zr_6_O_4_(OH)_4_(CO_2_)_12_ (Figure 3g). As the electron-rich π-conjugated system of the porphyrin-based ligand was extended, the light absorption range shifted from the visible spectrum to the NIR region. It was shown that TNP-MOF exhibited excellent photocatalytic CO_2_ reduction rates under near-infrared light (λ > 730 nm), reaching up to 6630 μmol·h^–1^·g^–1^. Moreover, the apparent quantum efficiencies (AQEs) of photocatalytic CO_2_ reduction of TNP-MOF were greater than 2.03% and 1.11% under 760 and 808 nm, respectively. This was also one of the highest AQEs under wavelengths greater than 750 nm among the NIR light-driven CO_2_ reduction photocatalysts reported so far. The preparation of these electron-rich photosensitive MOFs and the optimization of their photogenerated charge dynamics provided a new strategy for converting π-conjugated organic molecules to NIR-responsive MOF catalysts (Figure 3h,i). The anthracene-based linker was another effective electron-rich conjugated linker. Hou et al. embedded the electron transfer mediator (9,10-bis­(4-pyridyl)­anthracene (BPAN)) into the MOF cavity to construct a multilayer electron transfer pathway.^102^ By precisely adjusting the MOF cavity through the comparison of the number of guest molecules, BPAN-Co-2 showed a high CO photoreduction efficiency (5598 μmol·g^–1^·h^–1^), which was superior to that of most MOF-based catalysts.

Other linker functionalization strategies, such as phosphate functionalization,^103^ fluorination^104^ and covalent modification of MOF with different functional groups,^105^ have also demonstrated reduced band gaps and improved charge separation and transfer characteristics, which can enhance photocatalytic CO_2_ reduction performance. The photosensitizer functionalization and single-atom dispersed-site strategies will be discussed in detail in Sections 3.1.2 and 3.1.3.

Despite some progress in current research, the literature has indicated that linker functionalization is a viable and simple strategy to extend light absorption of organic ligands and enhance the separation and transfer of electrons. However, a number of non-negligible challenges remain, including achieving CO_2_ photoreduction under a wider range of conditions, enhancing catalytic efficiency, ensuring product selectivity, and evaluating the applicability of these catalytic systems on an industrial scale.

#### 3.1.2 Integrating Atomically Dispersed Metal Sites into Zr-MOFs

In recent decades, atomically dispersed metal sites (ADMSs) have garnered significant attention due to their high atomic utilization rate and unsaturated coordination configurations, which contribute to their exceptional catalytic performance. Researchers have devoted considerable efforts to designing and synthesizing ADMS catalysts supported on various substrates,^106–108^ with MOFs emerging as particularly promising candidates for ADMS loading.^109,110^ Specifically, ADMSs can be incorporated into pristine MOFs through adsorption at open metal sites postsolvent removal, encapsulation within the well-defined pores of MOFs, or even coordination with nitrogen-containing ligands.^111,112^ For instance, Wang et al. developed a photoreduction method to form single Cu atoms supported on UiO-66-NH_2_ supports (Cu-SAs/UiO-66-NH_2_), effectively enhancing the conversion of CO_2_ into liquid fuels.^113^ The Cu-SAs/UiO-66-NH_2_ exhibited enhanced light absorption and charge separation ability. The enrichment of photogenerated electrons altered the oxidation state of Cu atoms, inducing the activation of CO_2_ adsorbed into UiO-66-NH_2_, which significantly improved the photocatalytic efficiency of the conversion of CO_2_ to methanol and ethanol (Figure 4a). Jiang and his colleagues had developed a versatile and simple strategy for the construction of single-atom catalysts (SACs) with tunable coordination microenvironments.^114^ The Zr_6_-oxo clusters within MOFs (UiO-66-NH_2_) created neighboring −O/OH_*x*_ groups, which offered lone pair electrons and maintained charge balance, effectively immobilizing extrinsic Ni single metal atoms (Ni_1_-X/MOF). As a result, Ni_1_–S/MOF exhibited high photocatalytic activity due to enhanced light absorption, adjusted electronic states, and lowered proton activation barriers. The above studies provided new insights for the design of atomic-scale CO_2_ reduction photocatalysts, but the uncontrollable position and dispersion of single atoms within the MOF framework had an impact on the photocatalytic performance and mechanism studies.

**Figure 4:**
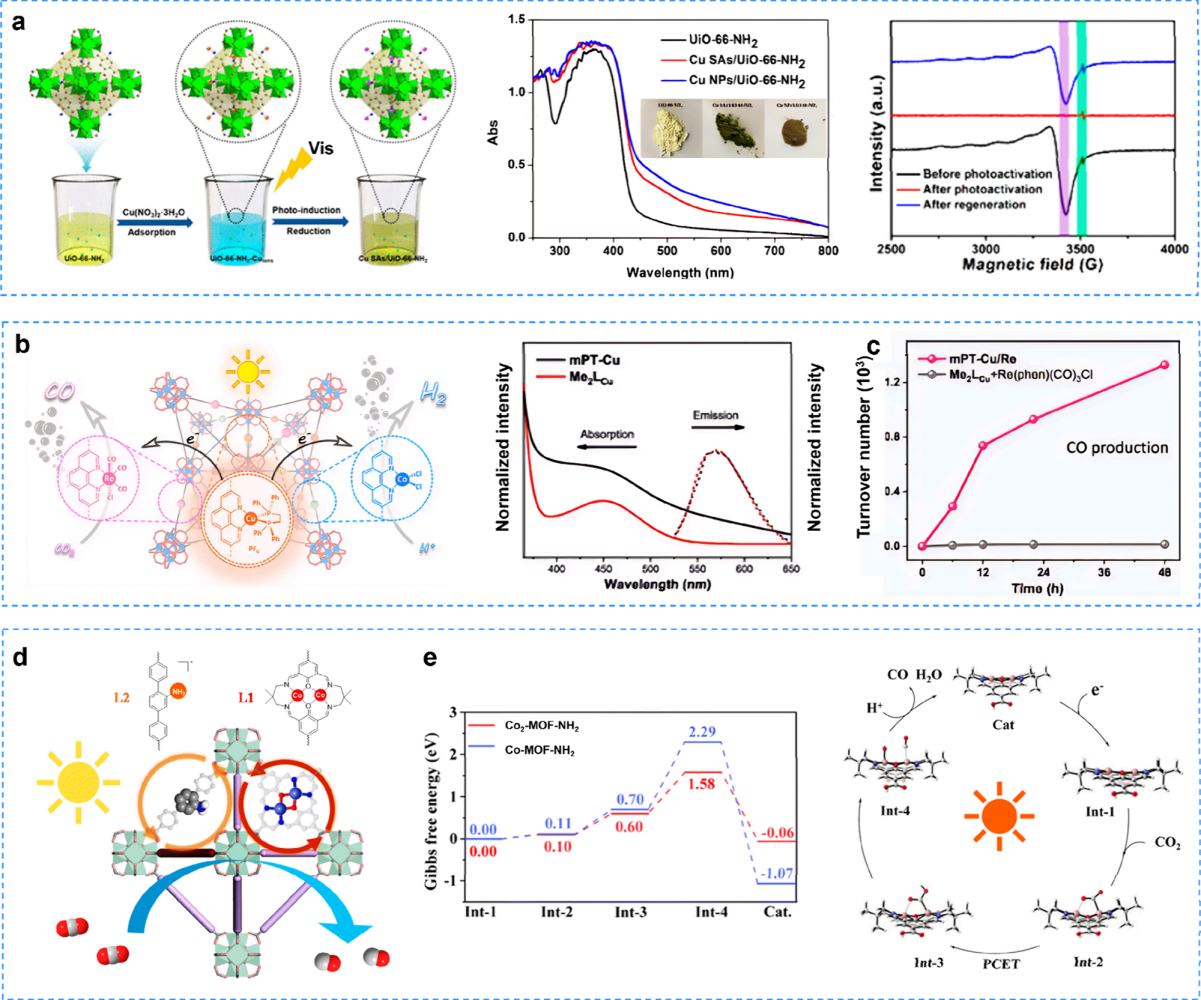
**a** Cu SAs/UiO-66-NH_2_ for photocatalytic CO_2_ reduction. Reproduced with permission from ref 113. Copyright 2020, American Chemical Society. **b** mPT-Cu/Re for CO_2_ photoreduction; **c** time-dependent CO_2_RR TONs of mPT-Cu/Re compared to the homogeneous control. Reproduced with permission from ref 116. Copyright 2020, American Chemical Society. **d** Framework structure and **e** catalytic cycle for the photocatalytic CO_2_RR of the Co_2_-MOF­(−NH_2_) binuclear catalyst. Reproduced with permission from ref 117. Copyright 2023, Royal Society of Chemistry.

The secondary metal active sites can be precisely introduced into the framework, which facilitates the tracing of reaction intermediates and investigation of the underlying mechanisms. For example, a particularly attractive approach involved integration of Ir, Re, and Ru complexes featuring dicarboxylic acid functional groups into the exceptionally stable and porous MOF UiO-67 using a mixed-ligand synthetic strategy.^115^ The resulting UiO-67 with single atom dispersed sites retains isostructural integrity with the inert pristine UiO-67 framework and exhibits high activity in photocatalytic CO_2_ reduction. Furthermore, the integration of a monodisperse Re catalytic center and Cu photosensitizer into MOFs (mPT-Cu/Re) through coordination with N in dibenzoic acid phenanthroline (PT) enhanced light absorption and facilitated multielectron transfer (Figure 4b).^116^ Under 350–700 nm light irradiation, the CO turnover number (TON) of mPT Cu/Re in a mixed solution of *N*,*N*-dimethylacetamide (DMA) and H_2_O was as high as 1328 (Figure 4c) with BIH as the sacrificial agent, which was nearly 2 orders of magnitude higher than the homogeneous control. In addition, luminescence quenching showed that electrons were transferred from lithium borohydride (BIH) to photoexcited Cu PS* to produce reduced Cu PS^–^, while electrons were injected into the Recatalytic site of the CO_2_ reduction reaction (CO_2_RR), triggering the photocatalytic CO_2_RR.

At present, most of the single-atom dispersed metal catalytic sites are noble metals such as Ir, Re, and Ru, which are expensive and unfavorable for practical applications despite their high catalytic efficiency. Designing and fabricating nonprecious metal photocatalysts with excellent photocatalytic performance and atomically precise structures using metal–organic frameworks (MOFs) remains a significant challenge. Inspired by the synergistic catalysis of biological enzyme systems, Lu et al.^117^ integrated highly active binuclear cobalt complexes into amino-functionalized MOFs, thus creating a synergistic diatomic catalyst (Figure 4d). A series of photoelectrochemical characterizations showed that the light absorption of the amino-functionalized catalyst was extended from 480 to 800 nm, and the CO_2_ adsorption capacity as well as the charge separation were improved. Using BIH as a sacrificial reagent, the Co_2_-MOF­(−NH_2_) binuclear catalyst exhibited excellent catalytic activity for the CO_2_RR with a CO yield of 2.44 mmol·g_Co_^–1^·h^–1^, which was 10.5 times higher than that of its mononuclear counterpart, CO-MOF­(−NH_2_). In situ FTIR and density functional theory (DFT) calculations indicated that COOH* exhibited stronger adsorption at the Co_2_ site due to the binuclear metal synergistic catalytic effect. (Figure 4e). The energy barriers further revealed the electron transfer and CO_2_ reduction reaction pathways. The Co center first accepted electrons from -NH_2_ to obtain intermediate products-1 (Int-1). Subsequently, Int-1 adsorbed CO_2_ to form the Co-CO_2_ adduct (Int-2). The Int-2 underwent the process of proton-coupled electron transfer (PCET) to produce Int-3. Following the addition of protons (H^+^) to form a water molecule, CO was released and catalyst was regenerated by an exothermic process.

The above studies show that the strong interaction of ADMSs with the Zr-MOF framework promotes the charge transfer between metal sites and framework. However, this presents significant challenges for catalyst design and remains unclear, in terms of the mechanisms of light absorption and electron transfer. Consequently, developing highly active and selective CO_2_ reduction photocatalysts and elucidating the catalytic mechanisms are the most critical and challenging.

#### 3.1.3 Anchoring a Photosensitizer in Zr-MOFs

Efficient photocatalysts can utilize visible light (400 nm < λ < 800 nm) as much as possible for photocatalytic reactions, since ultraviolet light constitutes only 5% of solar radiation. The photosensitizers can be introduced into appropriate nanospaces of MOFs via covalent grafting to enhance light harvesting and enable heterogeneous transformation of the photosensitizers due to the regularity of MOF structures and their tunable porosity. A series of photosensitizers, including vitamin B_2_ (VB_2_), subphthalocyanine (SubPc), and organic/inorganic optical dyes, have been successfully incorporated into MOF frameworks.^118–120^ These photosensitizers act as efficient light-absorbing antennas, transferring photoinduced electrons to the framework skeletons. The MOF composites anchored with photosensitizers can be categorized into two systems based on different connection methods. The first involves chemical covalent bonding, illustrated by examples such as VB_2_@UiO-66 ^121^ and EY@UiO-66-NH_2_,^122^ where −OH and —C=O groups within the MOF structure bind to unsaturated metal atoms. This approach ensures uniform immobilization and distribution of photosensitizers within the framework, enhancing electron and hole transfer. Lin et al. developed a novel synthesis method for bifunctionalized FeX@Zr_6_–Cu MOFs (X = Br^–^, Cl^–^, AcO^–^, or BF^4–^) by integrating subcopper photosensitizer (Cu PS) linkers with catalytically active Fe^II^ centers located at the μ_3_–OH sites of the Zr_6_(μ_3_-O)_4_(μ_3_–OH)_4_ SBU­(Fe).^123^ The close contact between the Cu PS and Fe sites enhances light absorption and facilitates rapid charge transfer, resulting in efficient photocatalytic activity (Figure 5a). Photoelectrochemical and photophysical characterization revealed an electron transfer pathway in the photocatalytic process. Upon the light irradiation, Cu PS* was reduced by BIH to form Cu PS-, subsequently transferred electrons to Fe^II^, generating Fe^I^ and initiating the proton reduction process. Maji et al. cografted the molecular photosensitizer (PS) Ru­(MBA)­(bpy)_2_Cl_2_ (bpy:2,2′-bipyridine) and the catalyst Mn­(MBA)­(CO)_3_Br inside Zr-MOF-808 (Zr-MOF) nanopores to obtain Zr-MBA-Ru/Mn-MOF for CO_2_RR.^124^ Without any additional electron-sacrificing reagents, Zr-MBA-Ru/Mn-MOF achieved a CO yield of up to 1027 μmol·g^–1^ and selectivity >99% after 26 h of CO_2_ reduction. The change of carbonyl stretching frequency of the Mn­(MBA)­(CO)_3_Br complex under light irradiation was monitored by in situ diffuse reflectance infrared Fourier transform spectroscopy (DRIFT) and DFT calculations to reveal the mechanism of photoinduced electron transfer from Ru­(II) to Mn­(I) (Figure 5b). In addition, *CO and *COOH species were monitored by in situ DRIFT, confirming the reaction pathway of CO_2_ → *CO_2_ → *COOH → *CO → CO. Alternatively, loading could be achieved through nonbonded π-π stacking and van der Waals interactions, exemplified by ErB#UiO-66 ^125^ (ErB = erythrobacterin B) and Pt-Calix-3#UiO-66-NH_2_.^126^

**Figure 5:**
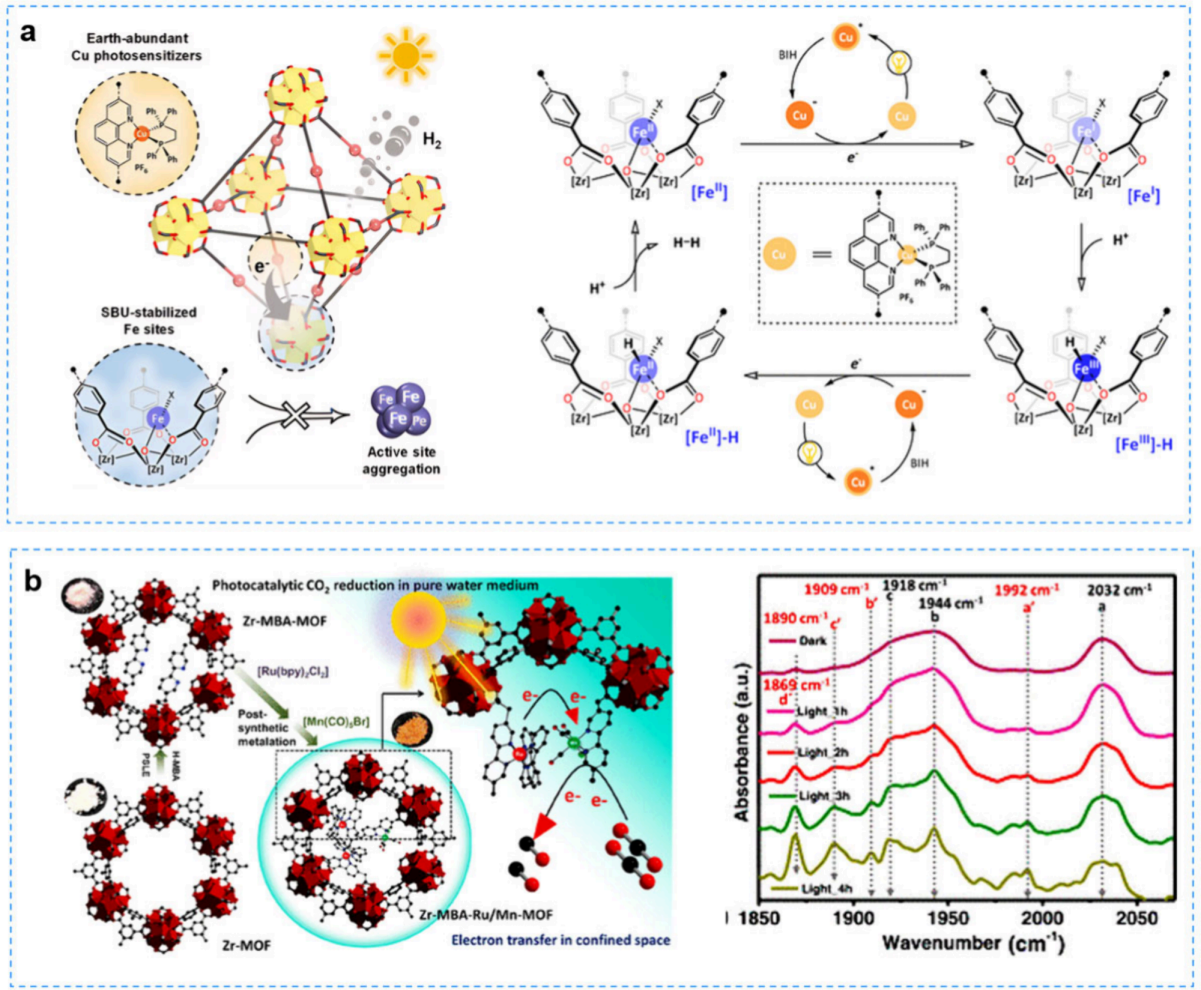
**a** The integration of Cu photosensitizers and secondary building-unit-supported Fe catalysts within Zr-MOFs for photocatalysis. Reproduced with permission from ref 123. Copyright 2020, American Chemical Society. **b** Confining molecular photosensitizer and catalyst in Zr-MBA-Ru/Mn-MOF for photocatalytic CO_2_ reduction and the mechanism of photoinduced electron transfer. Reproduced with permission from ref 124. Copyright 2023, American Chemical Society.

The anchoring of photosensitizers, especially some noble metal molecular photosensitizers, into the Zr-MOF framework can improve light absorption and promote recyclability. However, it remains unclear whether this anchoring improves catalytic efficiency compared to homogeneous catalytic mechanisms as well as the detailed reaction mechanisms involved. Therefore, the development of Zr-MOF heterogeneous catalysts with high activity and selectivity for coanchoring the photosensitizer and catalytic center continues to present significant challenges.

Other effective strategies include the introduction of defects to modulate the band gap of Zr-MOFs or other photosensitive components. For instance, the UiO-66/Co_9_S_8_ composite was engineered to synergize light absorption and CO_2_ adsorption advantages for CO_2_ to CH_4_ conversion under IR light irradiation.^127^ The finite element method simulations revealed that the metallic properties of Co_9_S_8_ enabled UiO-66/Co_9_S_8_ to have excellent IR light absorption capabilities, while UiO-66 significantly enhanced the local CO_2_ concentration. Consequently, neither Co_9_S_8_ nor UiO-66 demonstrated an observable IR photocatalytic activity. However, UiO-66/Co_9_S_8_ exhibited a remarkable performance. Under infrared light irradiation, UiO-66/Co_9_S_8_ achieved a CH_4_ release rate of 25.7 μmol·g^–1^·h^–1^ with nearly 100% selectivity, surpassing those of most reported catalysts. Additionally, a series of UiO-66-NH_2_ catalysts with missing linker (ML) or missing cluster (MC) defects and varying degrees of ligand vacancies have been successfully synthesized for photocatalytic CO_2_ reduction.^128^ Among these, UiO-66-NH_2_-ML-100, featuring ML defects, demonstrated a photocatalytic CO yield of 21.3 μmol·g^–1^·h^–1^, marking a 2.2-fold improvement compared to UiO-66-NH_2_-MC-150 with MC defects. The UV–visible diffuse reflectance spectra confirmed that ML UiO-66-NH_2_-ML-100 exhibited superior light absorption across both ultraviolet and visible light ranges.

### 3.2 Accelerated Photoinduced Carrier Separation and Transfer

Prolonging the lifetime of photogenerated carriers and suppressing electron–hole recombination have long been primary research objectives that dictate the efficacy of photocatalysts. The regulation of charge separation directly influences the lifetime of photoinduced charge carriers and can facilitate photocatalytic reactions.

#### 3.2.1 Constructing Z-Scheme or Heterojunction

Various strategies for enhancing charge transfer in Zr-MOF-based composites have garnered significant attention in research, particularly through the construction of Z-Scheme systems or heterojunctions. These approaches integrate a range of materials, including highly conductive substances such as semiconductors, graphene, and C_3_N_4_.^129–134^ as well as visible light-responsive components like photosensitizers.^135^ To simplify and clarify the electron transfer mechanisms within MOF-based materials, these processes are categorized into three types: Z-Scheme, Type I, and Type II heterojunctions are illustrated in Figure 6. Depending on the energy states of photoinduced electrons and holes within the photosensitizer (PS), migration occurs toward regions with lower reduction/oxidation potentials, specifically toward the conduction band (CB) and valence band (VB) of another PS. In Type I and Type II heterojunctions depicted, electrons move downward while holes move upward. The key distinction lies in whether reduction and oxidation reactions take place within a single component (Type I) or across two distinct components (Type II). The incorporation of a second component PS effectively enhances the spatial separation of photoinduced electrons and holes, thereby boosting photocatalytic activity.

**Figure 6:**
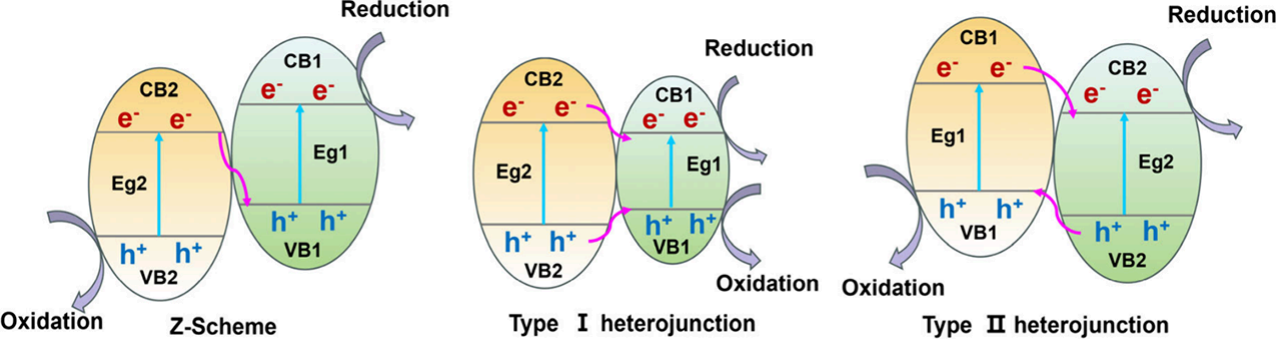
Schematic diagrams of charge transfer for Z-Scheme, type-I, and type-II heterojunctions within MOF-based materials.

A noteworthy example was the UiO-66/CNNS hybrid MOF loaded with carbon nitride nanosheets (CNNS),^136^ which was prepared through electrostatic self-assembly between negatively charged nano-CNNS and positively charged UiO-66. The long lifetime of electron–hole pairs and the high CO_2_ reduction performance could be attributed to the high electron mobility, enabled by the efficient transfer of electrons excited from CNNS to UiO-66 (Figure 7a). While both heterojunction types exhibited enhanced photocatalytic activity, they did not fully exploit the redox potentials of their components. This limitation had spurred the development of the Z-Scheme heterojunction, which optimized redox potentials and accelerated electron transfer rates. This acceleration was enabled by strong electrostatic interaction between electrons from PS-I and photogenerated holes from PS-II.

**Figure 7:**
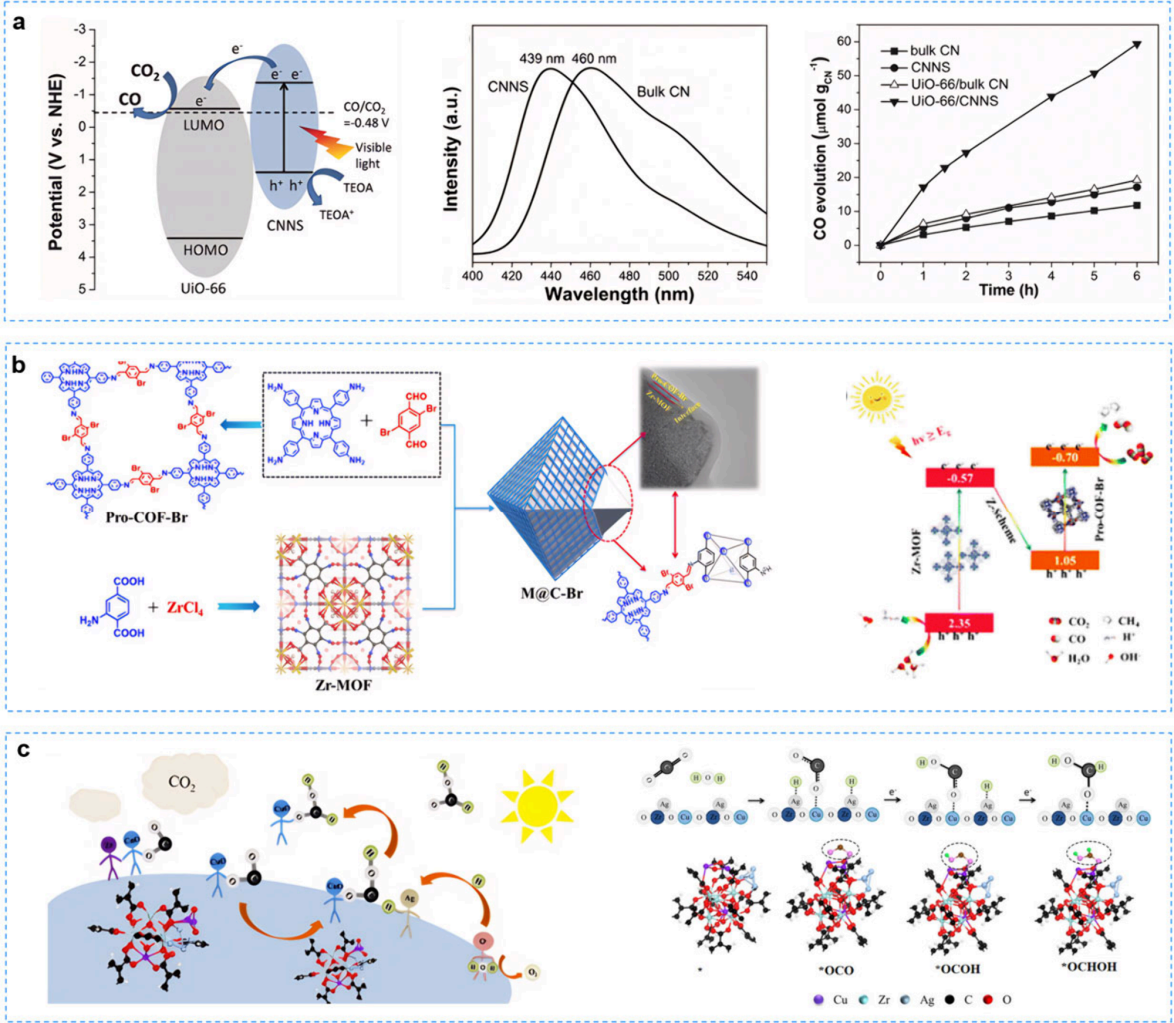
**a** UiO-66/carbon nitride nanosheet heterogeneous photocatalyst for CO_2_ reduction. Reproduced with permission from ref 136. Copyright 2015, Wiley-VCH. **b** The porphyrin-based core–shell Zr-MOF@Pro-COF-Br Z-Scheme heterojunction for efficient visible-light-driven CO_2_ reduction. Reproduced with permission from ref 137. Copyright 2023, Royal Society of Chemistry. **c** CuO/Ag/UiO-66 Z-Scheme heterojunction for highly selective photocatalytic CO_2_. Reproduced with permission from ref 138. Copyright 2022, Elsevier.

One compelling aspect of the Z-Scheme is that photogenerated electrons remain within the CB1 of PS-I, retaining a stronger reduction potential, while holes reside in the VB2 of PS-II, maintaining a stronger oxidation potential (Figure 6). This arrangement ensures that both electrons and holes are more effectively utilized in redox reactions. Within the context of Z-Scheme systems integrated into Zr-MOFs, the Zr-MOF@Pro-COF-Br system, reported in 2023,^137^ exhibited superior photocatalytic performance. The in situ encapsulation of Pro-COF-Br onto the NH_2_–UiO-66 core through the Schiff base reaction, constructing the Z-type heterostructured M@C–Br-1 photocatalyst that notably enhanced the efficiency of photogenerated electron–hole separation (Figure 7b). Among them, the Pro-COF-Br core exhibited high CO_2_ adsorption and visible light absorption. The C–N covalent bond formed at the interface enhanced the photogenerated electron transfer rate. As a result, M@C–Br-1 exhibited a high CO yield of 106.35 μmol·g^–1^, which was 2.6 and 3.2 times higher than that of Zr-MOF (40.65 μmol·g^–1^) and ro-COF-Br (33.21 μmol·g^–1^), respective, with a high CO/CH_4_ selectivity of 63.17%. Electron paramagnetic resonance (EPR) and ultraviolet electron spectroscopy (UPS) revealed electron transfer pathways within the M@C–Br-1 Z-type heterostructure. The Zr-MOF and Pro-COF-Br were photoexcited to produce electrons and holes driven by the built-in electric field and interfacial energy band bending. The excited electrons of Zr-MOF tended to migrate to Pro-COF-Br and recombined with holes on the VB at the heterojunction interface. Then the electrons generated by constant photoexcitation migrated to the CB of Pro-COF-Br. This work provided a facile and effective strategy for the construction of MOFs@COF Z-type heterojunction photocatalysts with an enhanced photocatalytic performance.

Cui et al.^138^ reported a CuO/Ag/UiO-66 Z-type heterojunction catalyst, in which CuO/UiO-66 acted as the base photocatalyst and Ag acted as a bridge to facilitate charge transfer and separation (Figure 7c). In the absence of any sacrificial agents, the selectivity of CuO/Ag/UiO-66 photocatalytic CO_2_ reduction for HCOOH production was as high as 95.9%, which may be the highest selectivity to date. Band structure results, in situ FT-IR combined with DFT elucidated the possible electron transfer pathways and catalytic mechanisms. The photogenerated electrons on UiO-66 were transferred to CuO via Ag nanoparticle bridges and recombined with the photogenerated holes left in the valence band. The photogenerated electrons in CuO and holes in UiO-66 were retained, acting as CO_2_ reduction sites to produce HCOOH and as water oxidation sites to provide proton *H, respectively. In addition, the Ag nanoparticles exhibited a strong adsorption capacity for *H protons, which promoted the highly selective production of HCOOH by coupling the adsorbed *H with neighboring *OCO adsorbed on CuO. This work provided a general approach to achieving product selectivity modulation using metal cocatalysts.

The effectiveness of the heterojunction strategy for photocatalytic CO_2_ reduction in Zr-MOF has been well demonstrated. This method can effectively enhance light absorption, charge separation, and transfer, thereby improving the efficiency and selectivity of photocatalytic CO_2_ reduction, particularly for C_2_ products. However, it is inevitable that the pores of Zr-MOFs will be blocked in the process of constructing heterojunction, which is also a point of attention and concern for the design of Zr-MOF heterojunction catalysts in the future.

#### 3.2.2 Integrating Metal Nanoparticles into Zr-MOFs

Doping of another conductor, metal nanoparticles, into MOFs represents a significant strategy for enhancing photogenerated carrier separation and transfer, thereby improving the photocatalytic efficiency. Although numerous studies have reported the loading of precious metal nanoparticles into MOFs and demonstrated enhanced carrier dynamics, challenges persist in catalyst synthesis and characterization. The key issues include the uniform distribution of nanoparticles within the frameworks, their localization (whether on the surface or partially within pores), and strategies to prevent nanoparticle aggregation and control the particle size. The stability and recyclability of the catalyst are also crucial, especially considering whether precious metal nanoparticles will leach into the reaction solution and convert to metal ions. Serre et al. first reported a room-temperature method for incorporating ultrasmall Cu nanoparticles (Cu NCs) into Zr-MOFs, achieving uniform dispersion without aggregation.^139^ Compared to Cu NCs confined within MOF pores, Cu NCs@MOFs core–shell composites exhibited enhanced photogenerated carrier separation and reactivity (Figure 8a). X-ray absorption spectroscopy (XAS) showed that the Cu in the composites was in the oxidation state of metallic Cu^0^, indicating the presence of ultrasmall Cu nanoparticles. The main products of photocatalytic CO_2_ reduction from CuNCs@MOFs core–shell composites under UV irradiation (385 nm) were CO and HCOOH. Additionally, the HCOOH selectivity can be modulated by varying the type of Zr-MOF (NCs@MOF-801 and NCs@UiO-66-NH_2_).

**Figure 8:**
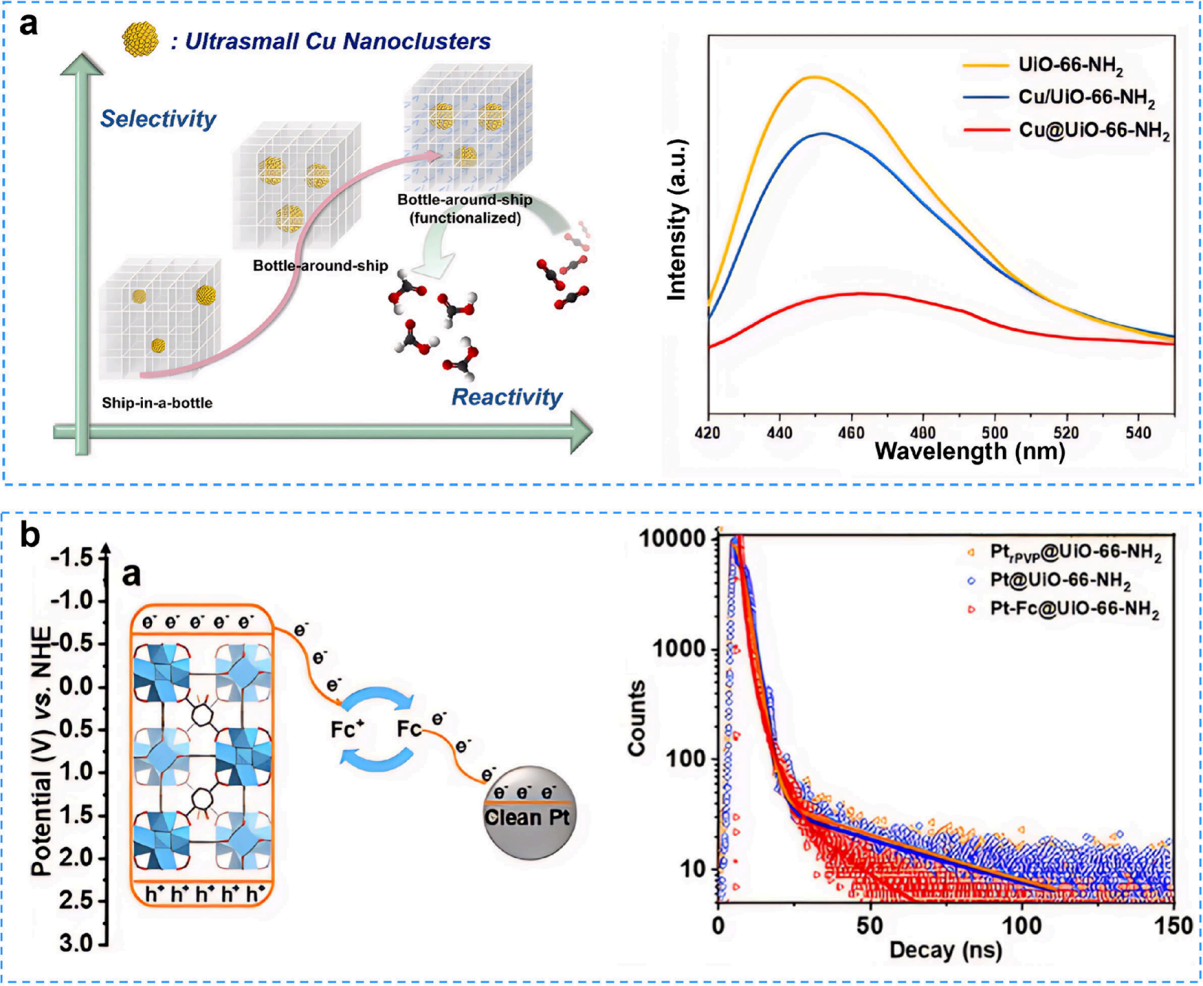
**a** The integration of ultrasmall copper nanoclusters in Zr-MOFs for CO_2_ photoreduction. Reproduced with permission from ref 139. Available under a CC-BY 4.0 license. Copyright 2022, The Authors, published by Wiley-VCH. **b** Enhancing photocatalysis through interfacial microenvironment modulation promotes electron transfer between Pt nanoparticles and MOFs. Reproduced with permission from ref 140. Copyright 2021, Wiley-VCH.

Similarly, a series of Pt nanoparticles were encapsulated into the metal–organic framework (UiO-66-NH_2_) to regulate the microenvironment, resulting in the development of Pt@UiO-66-NH_2_ for photocatalytic reactions.^140^ The interfacial electron transfer between Pt nanoparticles and the MOF on Pt@UiO-66-NH_2_ significantly improved photocatalytic performance (Figure 8b). The EPR revealed that all of the photocatalysts displayed a strong signal peak at *g* = 2.0023 under light, which was attributed to the chain-to-cluster electron transfer (LCCT) in the oxygen center of Zr oxygen clusters. Time-resolved PL spectra further indicated that the loading of Pt nanoparticles promoted more efficient charge separation. The complete spatial separation of the oxidation and reduction cocatalysts can enhance charge separation and catalytic reaction efficiency. Zhang et al. designed a heterostructured photocatalyst, Pt@NH_2_–UiO-66@MnO_*x*_ (PUM), with Pt and MnO_*x*_ as cocatalysts.^141^ Pt NP and MnOx NP dispersed inside and outside NH_2_–UiO-66, respectively, can effectively inhibit photogenerated electron and hole recombination. The EPR revealed the photogenerated charge transfer pathways at the interface of Pt NP, MnOx NP and NH_2_–UiO-66 in the PUM sample. Initially, NH_2_–UiO-66 generated electrons and holes under visible light irradiation. Then, electrons were preferentially trapped by Pt-NP in PUM and participated in the reduction reaction. Subsequently, the holes were transferred from the MOF to the MnO_*x*_ NP and participated in the oxidation reaction. This work provided an effective approach to accelerate the kinetics of both surface oxidation and reduction reactions by loading spatially separated metal nanoparticles on MOF.

Integration of metal nanoparticles within the Zr-MOF framework for photocatalytic CO_2_ reduction has significant advantages, such as fast charge separation and transfer, the access of high selectivity and generation of high-value products, such as C_2_ products. However, there are several issues that require attention. A major challenge is whether the metal nanoparticles on nanoparticle/MOF composites will block the pores of the MOF for efficient reaction and substrate diffusion. In addition, it is crucial to achieve precise localization and uniform dispersion of metal nanoparticles on the MOF for efficient charge transfer and catalytic mechanism studies.

#### 3.2.3 Structural Design

MOF-based materials typically exhibit diverse topological structures, morphologies, and crystal sizes, which can be effectively tailored by adjusting linkers, secondary building blocks, metal clusters and synthesis conditions.^142^ Different topologies, morphologies, or sizes of the same MOF exhibit distinct optical responses, which are beneficial for modulating the charges transfer to the active site. The typical examples include the MIL-53, MIL-68, MIL-88b, MIL-100, and MIL-101 series, which were composed of the same terephthalic acid and metal precursors, but exhibited distinctly different crystal shapes and sizes, leading to different catalytic activities.^143–147^ Another reported example was the Zr/Ce-UiO-66 hierarchical porous composite,^148^ obtained by partially substitution zirconium in the UiO-66 hexanuclear clusters with cerium and subsequent acid treatment or annealing. The single-shell hollow structure (SSHS) or double-shell hollow structure (DSHS) of Zr/Ce-iO-66 effectively regulated the charge transfer and improved the photocatalytic activity and catalyst stability. Peng et al. prepared nanocages consisting of metal–organic layers (MOLs) using zirconium-based UiO-66-(OH)_2_ as a precursor by a cosolvent method. And the UiO-MOL-Co photocatalysts were obtained by adding single-atom Co^2+^ sites to the Zr-oxo nodes of the metal–organic layers (MOL) cages.^149^ Charge dynamics studies showed that the unique nanocage morphology of UiO-MOL-Co including the two-dimensional nanosheet structure and surface hydroxyl groups facilitated the charge transfer and increased the Co^2+^ site exposure and local CO_2_ enrichment (Figure 9a). This enabled UiO-MOL-Co_0.99_ to exhibit the highest CO yield (7.74 mmol·g^–1^·h^–1^) with a selectivity of 97.1%. This work highlighted the tailoring of metal–organic framework (MOF) morphology and functionality to improve the conversion of photocatalytic CO_2_RR. The full utilization of catalytically active sites within MOFs is the key to improving their photocatalytic performance. Shi et al.^150^ explored the light absorption and participation of catalytic sites within 2D-MOFs and 3D-MOFs in catalytic reactions (Figure 9b). Two novel photocatalysts, 2D-MOF-Re in 2D nanosheets and 3D-MOF-Re in 3D blocks, were constructed from Zr_6_ clusters and tetracarboxyporphyrin (TCPP). The photocatalytic CO_2_ reduction experiments demonstrated that the CO turnover number (TON) of 2D-MOF-Re reached 27.8 within 6 h, which was 50 times higher than that of 3D-MOF-Re, based on the equivalent number of catalytically active sites. These results suggested that some catalytically active sites inside the 3D block MOF were inactive due to their inability to absorb light. The low dimension of 2D-MOF-Re promoted the direct coordination of the carboxyl group on the catalytic center Re­(bpdcy)­(CO)_3_Cl with the Zr_6_ cluster in the MOF. This study elucidated the great potential of the dimensional reduction approaches in photocatalyst design.

**Figure 9:**
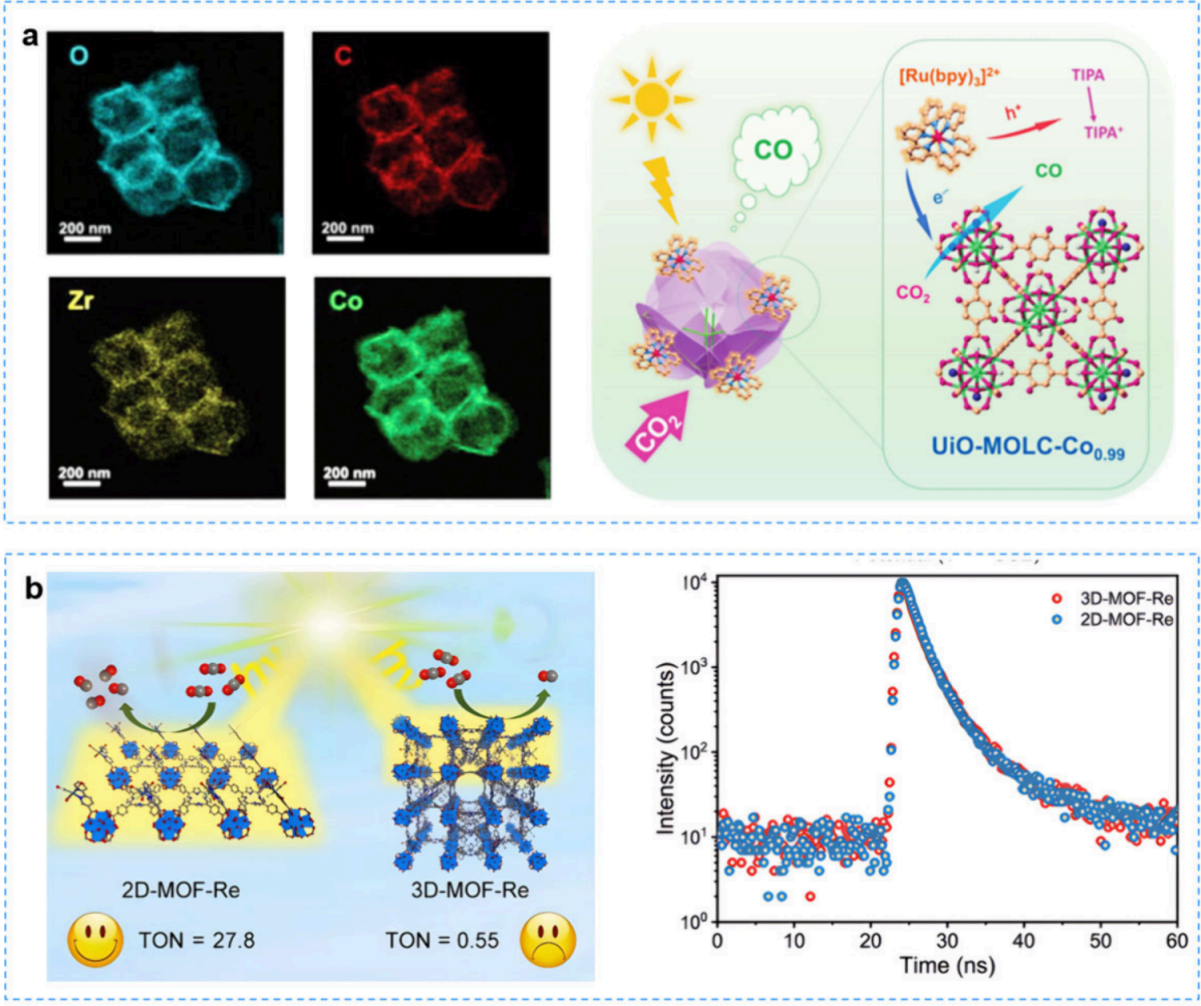
**a** Hydroxylated metal–organic-layer UiO-MOLC-Co_0.99_ nanocages for photocatalytic CO_2_ reduction. Reproduced with permission from ref 149. Copyright 2024, Springer. **b** Dimensional reduction enhances the photocatalytic CO_2_ reduction performance of 2D MOF-Re. Reproduced with permission from ref 150. Copyright 2024, Springer.

The structural design of Zr-MOF photocatalysts is a promising strategy to modulate the corresponding optical response and charge transfer properties by tuning the morphology, topology, crystal size, and dimension of the catalysts. Currently, there is no definitive evidence indicating which specific MOF structure has the best photocatalytic performance. However, the most favorable structural design principles to improve the optical performance of MOF materials are to increase the specific surface area and regulate the pore environment so that they can better utilize the visible light, facilitate the charge transfer, and expose more active sites.

Other effective strategies that can promote charge separation and transfer include the introduction of electron-rich components, modulation of mixed valence states in metal centers, and defect engineering.^151–155^ NH_2_–UiO-66-Fc was synthesized by incorporating electron-rich ligand ferrocene (Fc) into UiO-66, which facilitated the LMCT and therefore improved the photocatalytic CO_2_ reduction activity (Figure 10a).^156^ Theoretical calculations and multiple characterizations (photoelectrochemical characterization, in situ electron paramagnetic resonance, and fluorescence probe analysis) demonstrated that the LMCT energy of the MOF decreases with increasing Fc content, suggesting the existence of a dual-channel electron transfer mechanism. The 66-IS-M (M = Ni, Co, Cu) photocatalysts were developed by covalently anchoring isatin-Schiff base metal complexes (IS) within NH_2_–UiO-66 frameworks, immobilizing Ni, Co, and Cu ions into micropores, which exhibited notable CO_2_ photoreduction activity (Figure 10b).^157^ The systematic experiments and DFT calculations showed that effective charge separation in 66-IS-Ni and lowered free energy barriers for CO_2_ reduction, facilitating CO_2_ conversion. Pang et al.^158^ proposed the hybridization of PCN-222 with cellulose acetate (CA@PCN-222) through an optimized atomic interface strategy, which reduced the average valence state of Zr ions (Figure 10c). Experimental results demonstrated a significant improvement in the efficiency of electron transfer at the atomic interface between PCN-222 and cellulose.

**Figure 10:**
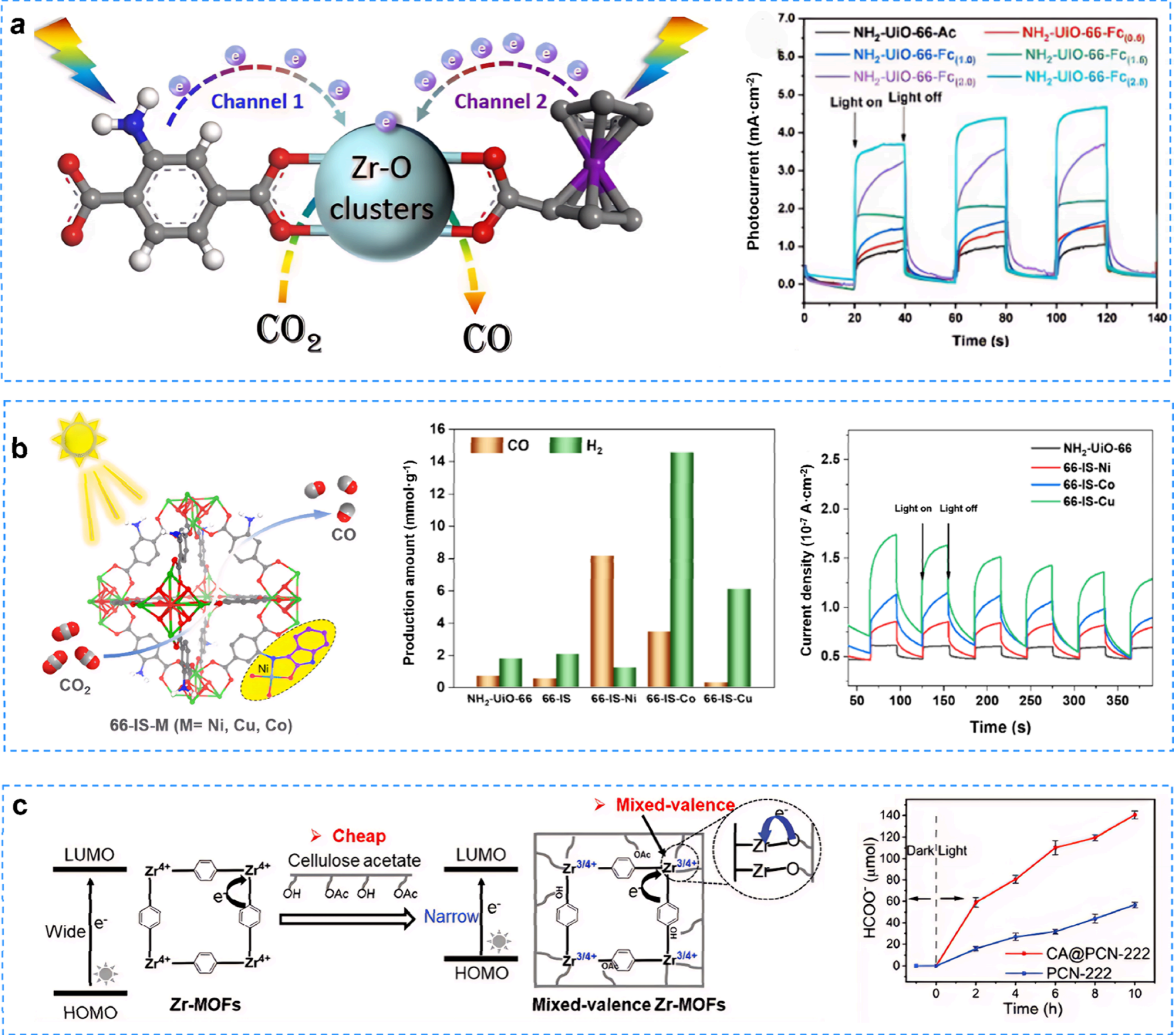
**a** The ferrocene-base Zr-MOFs for enhancing LMCT and frustrating Lewis acid in photocatalytic CO_2_ reduction. Reproduced with permission from ref 156. Copyright 2023, Elsevier. **b** The Isatin-Schiff base NH_2_–UiO-66 for highly photocatalytic CO_2_ reduction. Reproduced with permission from ref 157. Copyright 2023, American Chemical Society. **c** The formation of mixed-valence centers in Zr-MOFs for enhancing the photocatalytic Activity. Reproduced with permission from ref 158. Available under a CC-BY 4.0 license. Copyright 2023, The Authors, published by Wiley-VCH.

### 3.3 Enhanced Surface Redox Reactions

Many research efforts have primarily concentrated on enhancing the light absorption and carrier separation properties of photocatalysts, overlooking the critical factors of low CO_2_ absorption and activation, which significantly influence the CO_2_ reduction efficiency. Given that photocatalytic CO_2_ reduction typically occurs in aqueous environments, the competitive hydrogen evolution reaction directly impacts the selectivity of the photocatalytic process. The CO_2_ molecules are first adsorbed at the active sites of the catalysts. Subsequently, photogenerated electrons are transferred to the adsorption sites to activate the CO_2_ molecules. Finally, the active species and intermediates are desorbed for further reaction.^159^ The currently widely accepted possible structures of adsorbed CO_2_^.‑^ on catalysts include oxygen coordination, carbon coordination, and mixed coordination. Both oxygen and carbon atoms can donate electrons to the surface Lewis acid centers, and electron donors and acceptors can coexist within the CO_2_ molecule.^160,161^ The interactions among the CO_2_ molecules, active species, and surface atoms determine the reaction selectivity.

Recently, researchers have explored innovative catalyst designs to enhance visible-light-responsive photocatalytic CO_2_ reduction. For instance, modification of UiO-66 (Zr) with acetic acid (HAc) led to the synthesis of MOF catalysts with fully solid-state Lewis pair (FLPs)­Zr^3+^–OH.^162^ In-situ FTIR spectroscopy revealed that the activation of CO_2_ molecules was mediated by FLPs Zr^3+^–OH, resulting in the formation of b-CO_3_^2–^ (Figure 11a). The bonding orbital electrons of the CO_2_ gas molecules transferred to the vacant orbitals of the Lewis acid site Zr^3+^, while the lone pair electrons of the adjacent hydroxyl groups were transferred to the antibonding orbitals of the CO_2_. This led to the polarization of the CO_2_ molecule, the elongation of the molecular bond, and ultimately its dissociation. In 2021, Mei et al. constructed a biomimetic photocatalyst with flexible dual-metal site pairs (DMSP), integrating flexible Cu and Ni DMSPs into MOFs to obtain MOF-808-CuNi.^163^ During the photocatalytic process, Cu adsorbed carbon (C) from CO_2_ while oxygen (O) bounded with Ni, greatly stabilizing various C1 intermediates and thereby achieving a highly selective CO_2_-to-CH_4_ conversion process (Figure 11b). Additionally, Jiang et al.^164^ modulated CO_2_ adsorption/activation and charge separation capacity by adjusting the number of coordinating nitrogen atoms around single Co sites installed in UiO-type metal–organic frameworks (Figure 11c). Theoretical calculations showed that the Co sites in UiO–Co–N_2_ and UiO–Co–N_3_ were more readily bound to CO_2_ molecules compared to UiO–Co–N_4_. Consequently, UiO–CO-N_3_ exhibited a significantly enhanced CO production activity of 358.6 μmol/g, with a TOF of 1.025 h^–1^, which was 1.73 times higher than that of UiO–CO-N_2_.

**Figure 11:**
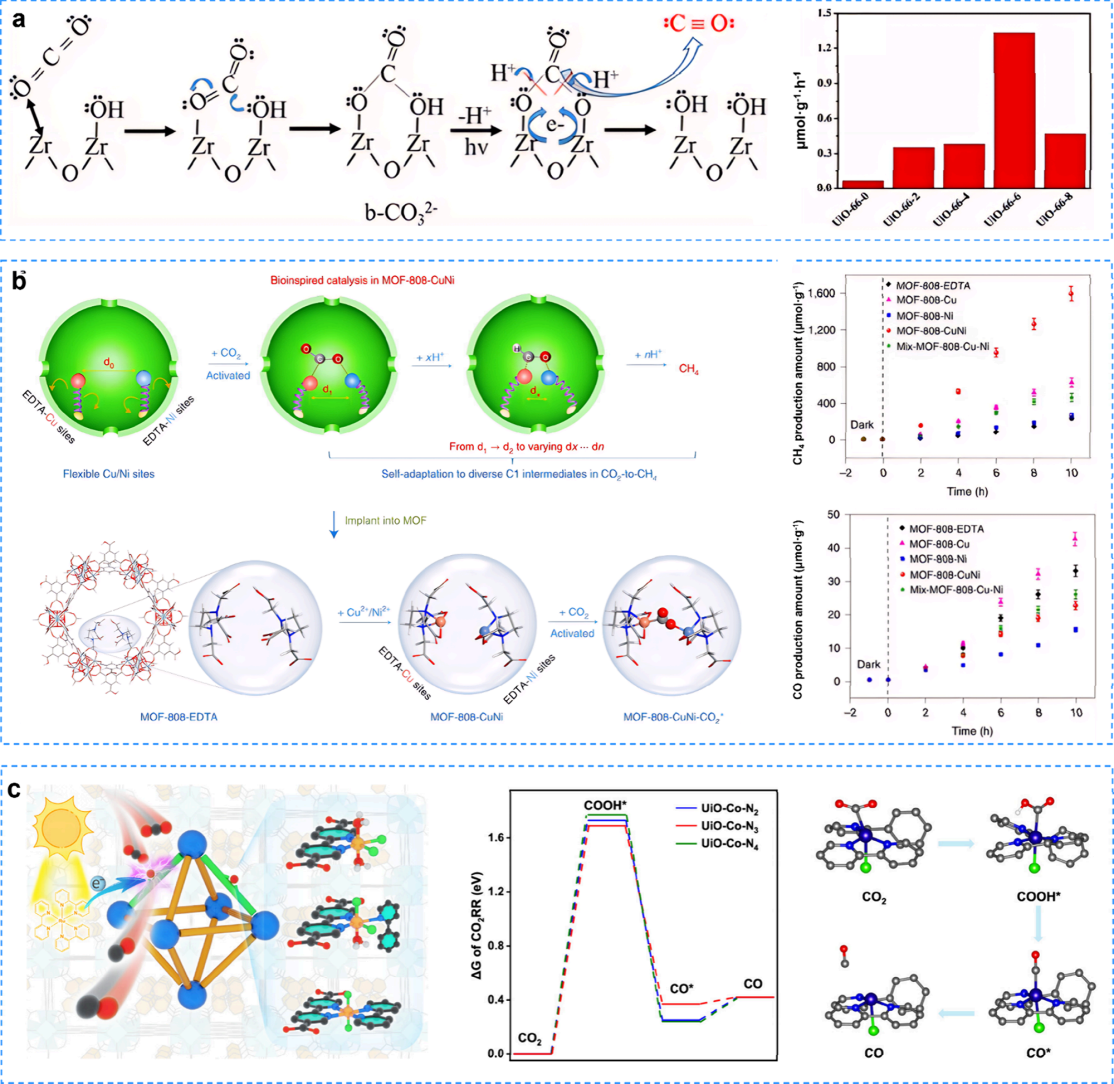
**a** UiO-66­(Zr) mediated with FLPs Zr^3+^–OH for efficient photocatalytic CO_2_ reduction by enhancing the activation of CO_2_ molecules. Reproduced with permission from ref 162. Copyright 2023, American Chemical Society. **b** The biomimetic Cu/Ni DMSPs photocatalysts for highly selective photoreduction of CO_2_ to CH_4_ through promoting the coordination of CO_2_ to Cu and Ni. Reproduced with permission from ref 163. Copyright 2021, Springer. **c** The regulation of coordinated N atom number around single Co sites in UiO–Co–N_2_ for boosting CO_2_ photoreduction. Reproduced with permission from ref 164. Copyright 2023, American Chemical Society.

In recent years, photochemical CO_2_ reduction has been extensively studied, and achieving high CO_2_ photoreduction activity in pure CO_2_ atmosphere. In contrast, only a few reports have been made for low concentrations of CO_2_ reduction. However, anthropogenic CO_2_ is mainly generated from industrial exhaust gases with CO_2_ concentrations of 5%–25%. The capture, purification, and concentration of CO_2_ in flue gases require substantial energy and capital investments. Therefore, the conversion of low-concentration CO_2_ or the direct use of CO_2_ from flue gas to produce valuable chemicals is an energy-efficient and cost-effective approach. Wang’s group^165^ developed TiO_2_/UiO-66 composites with hierarchical multilevel pore structures through a simple two-step synthesis, thus ensuring sufficient exposed catalytic sites and high CO_2_ uptake. As a result, the photocatalytic efficiency reached a level similar to that of pure CO_2_ atmosphere even under diluted CO_2_ conditions (≤2%). The photocatalytic enhancement mechanism suggested that the CO_2_ molecules were first concentrated in UiO-66 through the microporous network. Upon irradiation, the outer layers of the TiO_2_ nanoparticles were directly excited to generate electron–hole pairs. The interfacial interactions between TiO_2_ and UiO-66 promoted efficient separation of photogenerated electron–hole pairs. The designed composites demonstrated significant advantages in terms of the CO_2_ adsorption and exposure of catalytic sites, effectively improving catalytic performance under low-concentration CO_2_ conditions. However, the study of CO_2_ reduction at low concentration is still in its infancy and faces the challenge of poor reaction/conversion performance, which remains a key direction for future research.

In addition to the above methods, the development of Zr-MOFs with highly accessible active sites and the enhancement of the intrinsic activity of the MOFs are also a promising strategy to improve their photocatalytic CO_2_ reduction performance. For example, the introduction of spatially separated highly catalytic sites to reduce the CO_2_ activation energy can promote a surface redox reaction. Xu et al. prepared Ru single-atom-Cu nanoparticle bimetallic sites, which dramatically promoted the adsorption and activation capacity of CO_2_ molecules due to the strong interaction between Cu 3d and Ru 4d orbital.^166^ This effectively reduced the energy barrier for the rate-determining step (CO_2_ → COOH) of the reaction. The CO yield of Cu–Ru dual-site-modified Bi_4_Ti_3_O_12_ (BTO) was as high as 180.67 μmol·g^–1^·h^–1^ without any photosensitizer or sacrificial agent. This work provided a novel strategy for the design of photocatalysts for efficient photocatalytic CO_2_ reduction. Lu and his colleagues^167^ developed a DMC@cMOF-PVK film photocatalyst by leveraging the synergistic effects of steric hindrance and electrostatic driving strategies. The dual-metallic molecular catalyst (DMC) and perovskite (PVK) quantum dot photosensitizers were immobilized in the channels and on the surface of cMOF ultrathin films, respectively. The PVK photosensitizers enhanced light absorption and utilization, the periodic channel structure of Cu-CAT ensured the mass transfer of CO_2_ and H_2_O molecules, and the binuclear DMC improved the photocatalytic CO_2_ reduction efficiency. The DMC@cMOF-PVK exhibited a CO yield of 133.36 μmol·g^–1^·h^–1^ using H_2_O as an electron donor, which was much higher than that of PVK and DMC-PVK. This work facilitated the development of controllable integration of functional units into MOFs, and also opened up new avenues for the development of future photocatalysts.

The above studies indicate that enhancing the adsorption and activation of CO_2_, along with the construction of highly active catalytic sites, can improve the surface redox reactions in photocatalytic CO_2_ reduction. However, this strategy also faces problems, such as severe disconnection between CO_2_ adsorption sites and photogenerated electron aggregation sites. Additionally, the strong adsorption of the CO_2_ reduction products hinders the regeneration of reactive active sites. Therefore, it is essential to design and regulate the pore structure and surface microenvironment of Zr-MOF-based photocatalysts to address these issues and enhance the CO_2_ photoreduction efficiency.

In recent decades, photocatalytic CO_2_ reduction applications of Zr-MOFs have experienced unprecedented growth. However, large-scale photocatalytic CO_2_ reduction applications of Zr-MOFs and their derivatives have been far from being realized. Therefore, the improvement and innovation of preparation and modification strategies are needed to achieve breakthroughs in this field. We can design high-performance Zr-MOF photocatalytic CO_2_ reduction catalysts by focusing on the basic aspects that influence photocatalytic CO_2_ reduction and employing advanced characterization techniques to investigate and analyze in-depth electron transport and catalytic reaction mechanisms. Table 1 summarizes the various strategies and catalytic results to enhance the photocatalytic CO_2_ reduction efficiency of Zr-MOF in order to provide a comprehensive and clear understanding of the achievements made in this field and the obstacles that still need to be addressed.

**Table 1:** Summarized Recent Developments of Zr-MOFs for Photocatalytic CO_2_ Reduction

Photocatalyst	Irradiation	Condition	Reaction rate (products)	ref.
Zr-SDCA-NH_2_	λ ≥ 420 nm	40 mg, MeCN/TEOA (30/1)	96.2 μmol·h^–1^·mmol _MOF‑1_ (HCOOH)	^97^
aU(Zr/In)	λ ≥ 420 nm	15 mg, H_2_O, gas–solid reaction	37.58 ± 1.06 μmol·g^–1^·h^–1^ (CO)	^98^
TNP-MOF	λ ≥ 730 nm	MeCN, TEOA (50 mM)	6630 μmol·h ^–1^·g^–1^ (HCOOH)	^101^
CuSAs/UiO-66-NH_2_	λ ≥ 400 nm	0.1 g, H_2_O (50 mL), TEOA (100 μL)	5.33 (CH_3_OH), 4.22 μmol·h^–1^·g^–1^(C_2_H_5_OH)	^113^
Co_2_-MOF(−NH_2_)	Full spectrum	1 mg, MeCN/H_2_O (4/1), BIH (0.025 M)	2.44 mmol·g_Co_^–1^·h^–1^	^117^
Zr-MBA-Ru/Mn-MOF	λ ≥ 400 nm	1 mg, H_2_O (10 mL)	1027 μmol·g^–1^ (CO, 26 h)	^124^
UiO-66/Co_9_S_8_	λ ≥ 800 nm	5 mg, H_2_O, gas–solid reaction	25.7 μmol·g^–1^·h^–1^ (CH_4_)	^127^
UiO-66-NH_2_-ML-100	λ ≥ 400 nm	2 mg, H_2_O, gas–solid reaction, TEOA	21.3 μmol·g^–1^·h^–1^ (CO)	^128^
M@C–Br-1	300 W Xe lamp	10 mg, NaHCO_3_ (0.084), H_2_SO_4_ (0.3 mL, 2 M)	106.35 μmol·g^–1^ (CO)	^137^
CuO/0.04Ag/UiO-66	300 W Xe lamp	0.03 g, H_2_O (100 mL), 0.3 MPa	60 μmol·g^–1^·h^–1^ (HCOOH)	^138^
Cu NCs@MOFs	250–385 nm	10 mg, DMA (9.4 mL), TEOA (0.6 mL)	128 μmol·h^–1^·g^–1^ (HCOOH, 86%)	^139^
UiO-MOL-Co_0.09_	λ ≥ 420 nm	MeCN/H_2_O, TIPA, Ru(bpy)_3_^2+^	7.74 mmol·g^–1^·h^–1^ (CO, 97%)	^149^
2D-MOF-Re	300 W Xe lamp	10 μmol Re, CH_3_CN, TEOA, BIH	27.8 (TON, 6 h)	^150^
NH_2_–UiO-66-Fc_(2.0)_	400–800 nm	2 mg, H_2_O (1 mL), MeCN (8 mL), TEOA (1 mL)	90.65 μmol·h^–1^·g^–1^ (CO)	^156^
66-IS-Ni	λ ≥ 400 nm	30 mg, H_2_O (4 mL), MeCN (60 mL), TEOA (4 mL), Ru(bpy)_3_Cl_2_·6H_2_O	1350 μmol·h^–1^·g^–1^ (CO, 87%)	^157^
CA@PCN-222	400–800 nm	50 mg, MeCN/TEOA (30/1)	2816.0 μmol·g^–1^ (HCOOH)	^158^
UiO-66-6	λ ≥ 420 nm	20 mg, H_2_O, gas–solid reaction	1.33 μmol·h^–1^·g^–1^ (CO)	^163^
MOF-808-CuNi		25 mg, MeCN/TEOA/H_2_O (3/1/1), (50 mg, 1.3 mM) Ru(bpy)_3_Cl_2_·6H_2_O	158.7 μmol·h^–1^·g^–1^ (CH_4_, 97.5%)	^164^

## 4 Advanced Characterization Techniques

In efficient photocatalytic reactions, advanced characterization techniques play a crucial role in elucidating processes such as light capture, charge separation, and surface redox reactions. These techniques are particularly vital, given the atomically precise and adjustable structures of metal–organic frameworks, which offer an exceptional opportunity to gain a clear understanding of photocatalytic mechanisms. This section first introduces time- and space-resolved techniques to investigate the dynamics of charge reactions in MOFs photocatalysis. Additionally, X-ray photoelectron spectroscopy, electron paramagnetic resonance spectroscopy, and diffuse reflectance infrared Fourier transform spectroscopy are discussed for monitoring photocatalytic reaction intermediates and elucidating related mechanisms.

### 4.1 Transient Absorption Spectroscopy

Photoelectrochemical characterizations, such as transient photocurrent, electrochemical impedance spectroscopy, and photoluminescence, provide insights into the separation and transfer dynamics of photogenerated charge carriers. However, the time resolution of electrochemical techniques typically spans from microseconds to seconds. In contrast, transient absorption spectroscopy (TAS) employs a pump–probe method where the photocatalyst is excited by pump pulse of light, and subsequent relaxation processes are monitored using probe pulse of light. The TAS offers time resolutions of absorption spectra ranging from femtoseconds (fs) to nanoseconds (ns) depending on the variation of the pulsed laser. Recently, ultrafast TAS on the femtosecond scale has emerged as a powerful tool to study photocatalytic charge transfer, energy transfer, and excited state lifetime in MOFs.

Incorporating electron-rich groups, such as −NH_2_, into organic linkers of MOFs represents a common strategy to broaden their light absorption capabilities. As a representative example, Xing and his co-workers synthesized a series of defective Zr-MOF-X (X = 160, 240, 320, or 400) by acid-modulated defect engineering and investigated their charge separation dynamics using ultrafast TAS.^168^ Electrochemical tests revealed that defective Zr-MOF-X had a more negative reduction potential and higher photocurrent response signal than did pristine NNU-28. Therefore, the defective Zr-MOF-X exhibited enhanced efficiency of photoreduction of CO_2_ to generate formate. The transient absorption spectra indicated that the structural defects modulated the excited state behavior and photogenerated charge separation of Zr-MOF-X, resulting in a slower decay process compared with the defect-free Zr-MOF. (Figure 12a). The long-lived Zr­(III) species in the defective Zr-MOF-X was fully exposed to a high CO_2_ concentration atmosphere, which improved the photocatalytic efficiency for CO_2_ reduction. Maji and his co-workers confined the π-hairpin and tetraphenyl (TET) molecules within the nanospace of postmodified Zr-MOF-808 (Zr-MBA-TET-Re-MOF) to efficiently utilize its light-trapping properties for photocatalytic CO_2_ reduction.^169^ Time-resolved photoluminescence (TRPL) decay demonstrated a significant increase in the average excited-state lifetime of Zr-MBA-TET-MOF (12.79 ns) compared with that of TET in DMF (4.33 ns) (Figure 12b). When the TET was encapsulated within the MOF, the confinement effect provided by the MOF easily suppressed the collision and diffusive relaxation of charges, which hindered the nonradiative decay pathway and significantly improved the fluorescence lifetime. Furthermore, the electron transfer pathways were inferred from in situ UV–vis, EPR, and TAS. The EPR signals at *g* = 1.99 and 2.004 could be attributed to the TET radical cation and one-electron reduced Re­(MBA^–^)­(CO)_3_Cl^–^ species, respectively, suggesting the electron transfer from TET to Re­(MBA)­(CO)_3_Cl.

**Figure 12:**
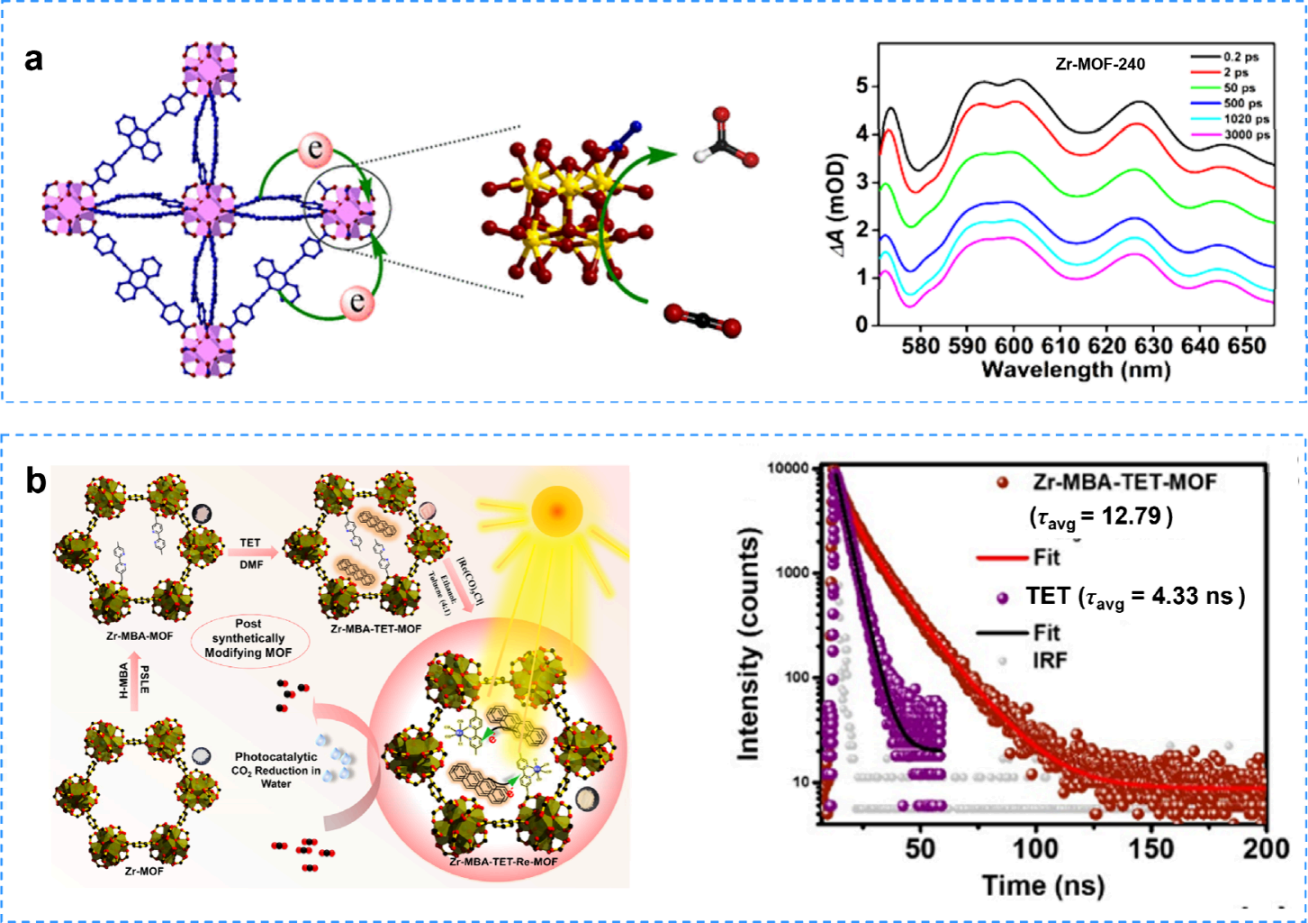
**a** The defective Zr-MOF-X for photocatalytic CO_2_ reduction and transient absorption spectra. Reproduced with permission from ref 168. Copyright 2024, American Chemical Society. **b** The photocatalytic CO_2_ reduction and TRPL of Zr-MBA-TET-Re-MOF. Reproduced with permission from ref 169. Copyright 2024, American Chemical Society.

Additionally, Jiang et al. successfully constructed asymmetric CuNi heteronuclear diatoms (CuNi-HDAs) by immobilizing single Cu atoms at the Ni sites within the (Ni,Zr)-UiO-66-NH_2_ framework.^170^ To further elucidate the dynamic charge transfer mechanism within Cu-(Ni,Zr)-UiO-66-NH_2_, fs-TAS spectroscopy was conducted using an excitation wavelength of 400 nm, as illustrated in Figure 13. Notably, the charge transfer was significantly enhanced due to the extension of the intramolecular charge transfer bands from Cu-(Ni,Zr)-UiO-66-NH_2_ to UiO-66-NH_2_ and (Ni,Zr)-UiO-6-6-NH_2_, as shown in Figure 13a, d, and g. It was noteworthy that the average lifetime of Cu-(Ni,Zr)-UiO-66-NH_2_, measuring 323.6 ps, was significantly shorter compared to that of UiO-66-NH_2_ (580.2 ps) and (Ni,Zr)-UiO-64-NH_2_ (422.0 ps) (Figure 13c, f, and (i), indicating effective charge transfer between (Ni,Zr)-UiO-66-NH_2_ and CuNi HDAs. This observation correlated well with the enhanced photocatalytic performance of Cu-(Ni,Zr)-UiO-66-NH_2_.

**Figure 13:**
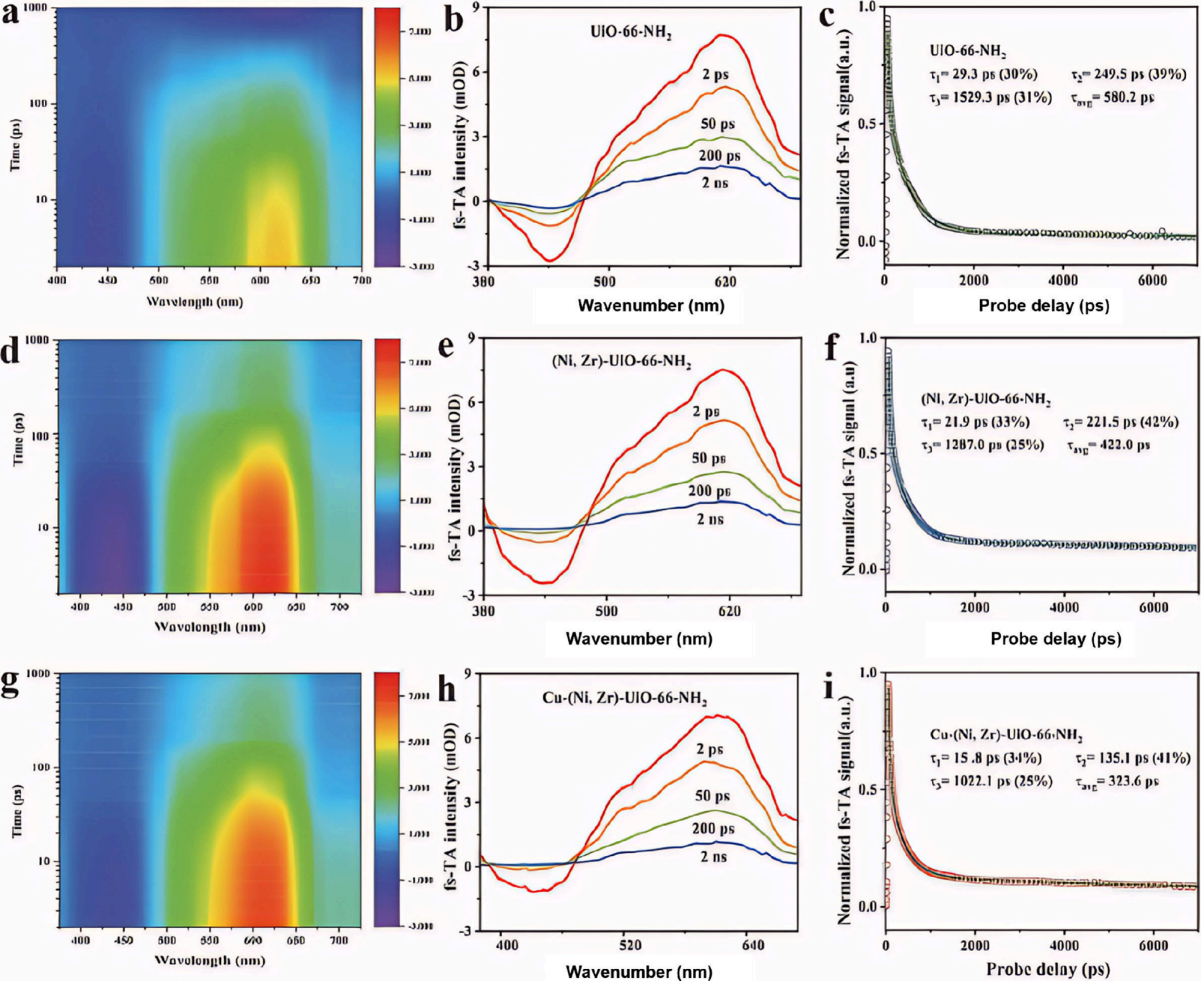
**a–c** 2D mapping of TA spectra, TA spectra signals, and normalized decay kinetic curves of UiO-66-NH_2_; **d–f** (Ni,Zr)-UiO-66-NH_2_; and **g–i** Cu-(Ni, Zr)-UiO-66-NH_2_. Reproduced with permission from ref 170. Copyright 2024, Elsevier.

### 4.2 X-ray Absorption Spectroscopy

In order to clarify the correlation between the evolution of the electronic structure of catalytic centers and the dynamics of charge transfer, and to precisely confirm the direction of charge transfer, the X-ray absorption spectroscopy (XAS) was performed combined with TAS techniques.^171–173^ A typical example was the CoRu-UiO-67 photocatalytic system, where intermediate species and reaction kinetics were revealed through in situ XAS and fs-TAS.^174^ Initially, fs-TAS confirmed the electronic separation and transfer process from the Ru complex to the Co site, as depicted in Figure 14a. To investigate the correlation between the evolution of the electronic structure of Co and electron transfer, in situ XAS was carried out to gather structural insights into the Co intermediates in photocatalytic reaction. The in situ X-ray absorption near-edge structure (XANES) spectra revealed significant red shifts in both region I (the edge) and region II (oscillations above the edge), as presented in Figure 14b. These shifts toward lower energy edges were indicative of the reduction of Co^2+^ to Co^+^, while the changes in the above-edge oscillations suggested a strengthening in the Co-L bond (where L represents the coordinating atom). Importantly, the altered XANES spectra reverted to their original state upon cessation of light exposure, thus ruling out catalyst degradation as the cause of these signal changes. Furthermore, Fourier-transformed X-ray absorption fine structure (XAFS) spectra provided confirmatory evidence that the reduction of Co^2+^ to Co^+^ was attributed to the expansion of Co–L to Co–N bonds (from 1.98 to 2.11 Å) and Co–Cl bonds (from 2.25 to 2.28 Å) within the first coordination shell, as depicted in Figure 14c. This comprehensive study revealed the formation of long-lived Co^+^ intermediates during electron transfer from Ru photosensitizer to the Co sites in photocatalysis. Moreover, Wang and colleagues^175^ demonstrated electron transfer from porphyrin organic linkers to single Pt catalytic centers during the photocatalytic process in a single-atom Pt anchored zirconium porphyrin MOF hollow nanotubes (HNTM-Ir/Pt), employing XAS and related photoelectric characterization (Figure 14d).

**Figure 14:**
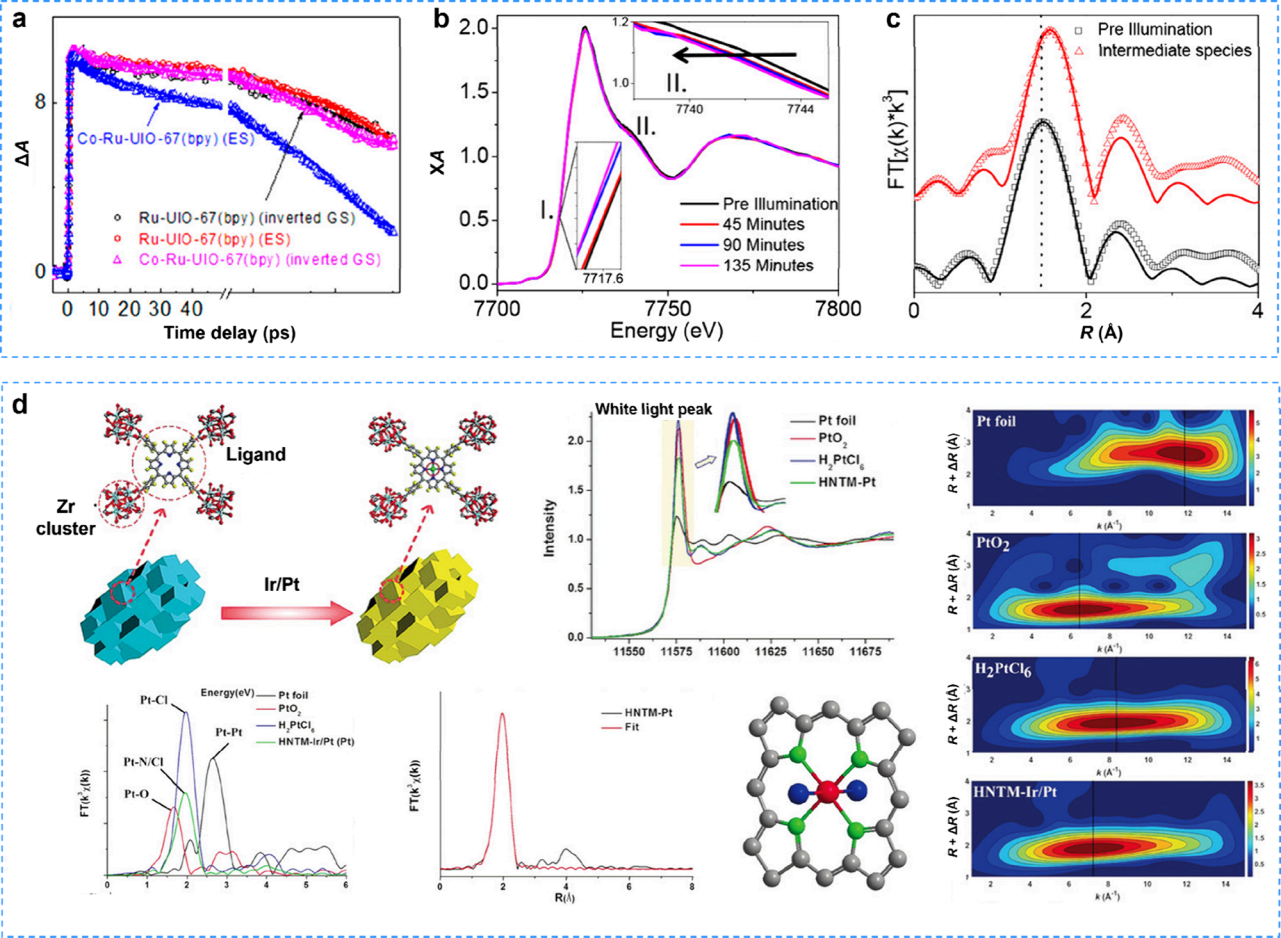
**a** GSB recovery and ES decay kinetics of Ru-UIO-67­(bpy) and Co–Ru–UIO-67­(bpy); **b** in situ XANES spectra of Co–Ru–UIO-67­(bpy) under dark and light; and **c** EXAFS spectra of Co–Ru–UIO-67­(bpy) under dark and light. Reproduced with permission from ref 174. Copyright 2018, American Chemical Society. **d** Synthesis scheme and XAS spectrum of HNTM-Ir/Pt. Reproduced with permission from ref 175. Copyright 2018, Wiley-VCH.

### 4.3 Diffuse Reflectance Infrared Fourier Transform Spectroscopy

Diffuse reflectance infrared Fourier transform spectroscopy (DRIFTS) is a widely used spectroscopic technique for materials characterization, where physical and chemical information about samples is obtained by measuring the reflected light intensity and frequency variations. It is a key tool for identifying intermediates involved in carbon dioxide reduction reactions to elucidate potential catalytic mechanisms by collecting diffusely scattered signals of infrared radiation on powders. Maji et al. investigated the photoreduction mechanism of CO_2_ to CO over Zr-MBA-Ru/Re-MOF.^176^ In the reaction system of CO_2_ and H_2_O, monodentate carbonate groups (m-CO_3_^2–^) emerged at 1314 and 1509 cm^–1^, resulting from the reaction between CO_2_ and H_2_O. Under photoirradiation, a significant intermediate in the CO_2_ to CO conversion, designated as COOH*, exhibited a gradual increase in intensity at 1620 cm^–1^, concurrent with the formation of CO* at 2060 cm^–1^ (Figure 15a). Based on these observed intermediates, a putative photocatalytic CO_2_ reduction pathway was delineated (Figure 15b). Under visible light irradiation, f-MOF-MBA-Ru^II^(bpy)_2_Cl_2_ absorbed visible light to form photoexcited f-MOF-MBA- Ru^III^(bpy^–^)­(bpy)^2+*^. The holes on photoexcited f-MOF-MBA-Ru^III^(bpy^–^)­(bpy)^2+*^ were quenched by water reduction, and electrons were transferred to f-MOF-MB-Re^I^(CO)_3_Cl. Finally, f-MOF-MBA-Re^I^(CO_2_)_3_ was formed after removing of Cl^–^. This process initiated the CO_2_ catalytic cycle supported by Re^I^. To gain a deeper and more comprehensive understanding of the photocatalytic reduction of CO_2_, in situ DRIFT spectroscopy was applied to detect the intermediates of CO_2_ conversion on HTiNbO_5_/UiO-66 (UTi) composite materials.^177^ As illustrated in Figure 15c, during the catalytic reaction of UTi-3 in the CO_2_ and H_2_O reaction system, several intermediates were observed to form, including monodentate carbonates (m-CO_3_^2–^ at 1293 and 1510 cm^–1^), bidentate carbonates (b-CO_3_^2–^ at 1277, 1337, and 1570 cm^–1^), and bicarbonate (HCO_3_^–^ at 1420 cm^–1^). Importantly, the peak intensities of m-CO_3_^2–^, b-CO_3_^2–^, and HCO_3_^–^ gradually intensified as the reaction progressed, which indicated that formate intermediates played a key role in the photocatalytic conversion of CO_2_ to CO. Furthermore, the in situ FTIR, photoelectric characterization combined with XPS showed that the interfacial interactions between UiO-66 and HTiNbO_5_ facilitated the transfer of electrons from UiO-66 to HTiNbO_5_, which subsequently reacted with CO_2_ molecules adsorbed on the catalyst surface to produce CO (Figure 15d). Guo et al.^178^ synthesized the Ce/Zr-IO-66-NH_2_/CdIn_2_S_4_ (Ce-NU66/CIS) bimetallic heterojunction via an in situ hydrothermal method and employed in situ FTIR techniques to monitor key intermediates during CO_2_ photoreduction to investigate the reaction pathway of CO_2_RR. The peaks at 1533, 1561, and 1649 cm^–1^ were attributed to the COOH* group, a critical intermediate commonly recognized for CO and CO_3_^2–^ formation.^179^ The peaks detected at 1063, 1114, and 1734 cm^–1^ were associated with CHO*, CH_2_O*, and CH_3_O* groups, respectively (Figure 15e). These intermediates played a crucial role in CH_4_ production, confirming the formation of both CO and CH_4_. Based on the observed reaction intermediates detected by FTIR combined with photoelectric characterization, the electron transfer pathway and reaction mechanism during the photoreduction of CO_2_ catalyzed by 8-Ce_0.2_NUC could be inferred (Figure 15f). Under light irradiation, the photogenerated electrons in the CB of Ce-NU66 transferred to the VB of CIS, and this unidirectional flow of electrons and holes hindered the electron–hole recombination in CIS. The photoexcited electrons in the conduction band were reduced by Ce^4+^ to form Ce^3+^, which combined with h^+^ to generate Ce^4+^. Thus, the unstable Ce^3+^ in Ce-NU66 led to Ce^3+^/Ce^4+^ cycling in the optimal composites, resulting in 8-Ce_0.2_NUC exhibiting excellent stability and efficiency.

**Figure 15:**
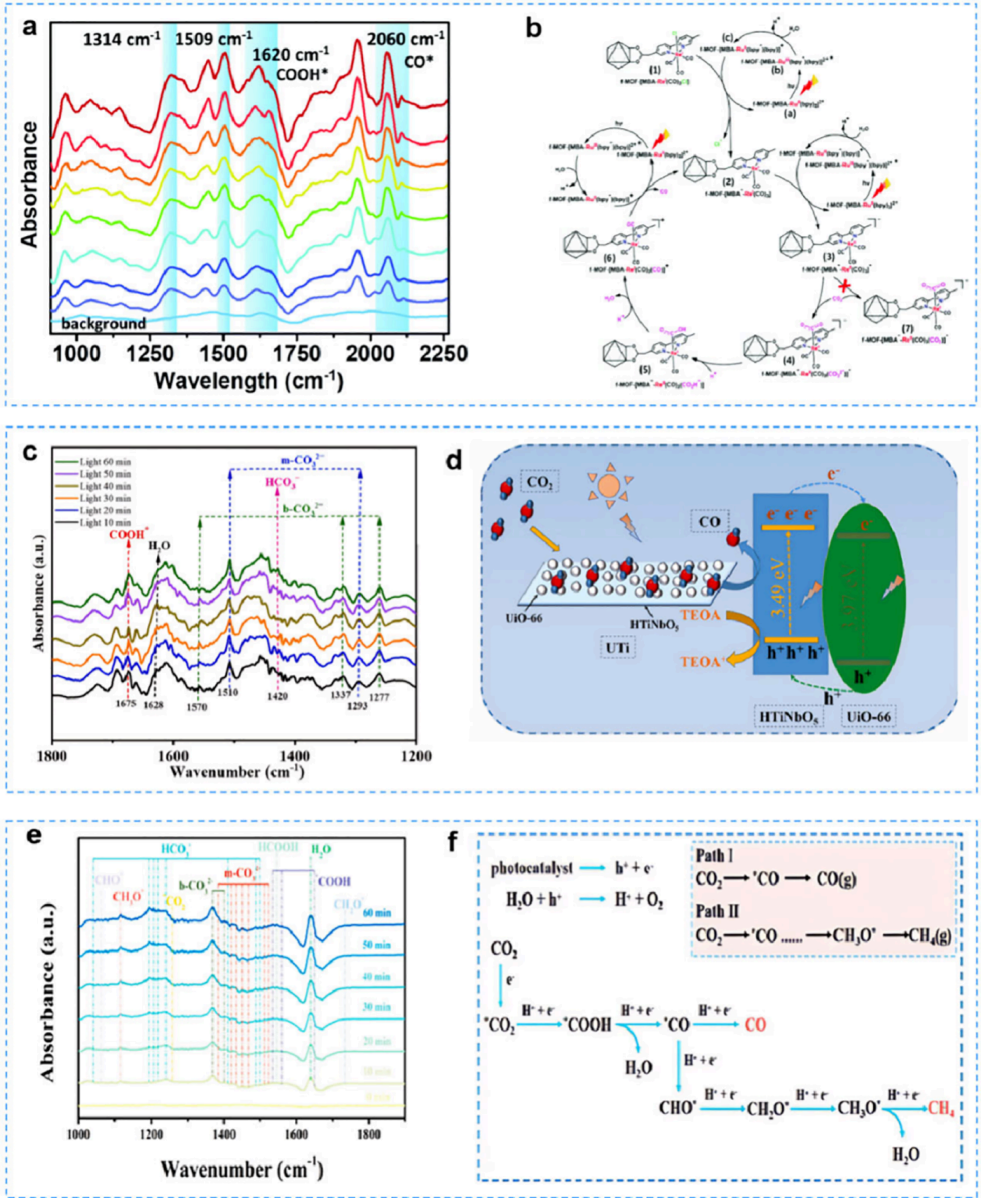
**a** The in situ diffuse reflectance FT-IR spectra; **b** the proposed reaction mechanism for CO_2_ reduction on Zr-MBA-Ru/Re-MOF. Reproduced with permission from ref 176. Copyright 2021, Royal Society of Chemistry. **c** In situ FTIR spectra; **d** the illustrative diagram of photocatalytic CO_2_ reduction mechanism for UTi-3 composite. Reproduced with permission from ref 177. Copyright 2023, Elsevier. **e** In situ FT-IR; **f** photocatalytic reaction pathways of 8-Ce_0.2_NUC. Reproduced with permission from ref 178. Copyright 2023, Elsevier.

Although DRIFTS is capable of capturing intermediates in CO_2_ photoreduction reactions, it is essential to note that not all intermediates identified in DRIFTS spectra necessarily participate in subsequent reactions.^180^ Therefore, a reliable elucidation of the reaction mechanism should rely on a comprehensive analysis that integrates DRIFTS results with other relevant data.

### 4.4 Electron Paramagnetic Resonance Spectroscopy

Electron paramagnetic resonance (EPR) spectroscopy is a powerful technique for detecting the presence of unpaired electron radicals in paramagnetic substances. This spectroscopic method relies on resonant transitions between electron spin energy levels within a static magnetic field, providing exceptional sensitivity to the geometric configuration and electronic structure of the paramagnetic substances. Notably, paramagnetic substances include transition metal ions and reactive radicals that play crucial roles in catalytic reactions, which can be monitored by EPR spectroscopy.^119^

The photocatalytic process of MOFs is accompanied by the appearance of many transient paramagnetic signals originating from reaction intermediates and the gain and loss of electrons for organic linkers and metal nodes. The *g*-factor (*g*-value) is a physical quantity that describes the ratio between an electron’s spin magnetic moment and its orbital magnetic moment. When a *g*-factor close to 2.003 is observed, this is usually reasonably attributed to free electrons since this value is nearly identical with the *g*-factor of a free electron. Analysis of these transient signals offers valuable insights into the charge transfer mechanisms and catalytic pathways. As an illustrative example, Jiang et al.^181^ developed PCN-222, a porphyrin-based MOF, to investigate photocatalytic reactions, accurately determined the LCCT process using EPR spectroscopy. Upon illumination, EPR spectroscopy detected heightened signals of porphyrin π-cation radicals within PCN-222 (Figure 16a), and it elucidated the directionality of electron transfer within its ligands. Notably, a distinct EPR signal at *g*_iso_ = 2.0021 emerged and intensified with prolonged irradiation (Figure 16b), suggesting that the electron transfer occurred between the Zr oxygen clusters and O_2_, leading to the generation of O^2̇‑^ as an active oxygen species. These findings collectively indicated that photogenerated electrons were transferred from the porphyrin ligands to the Zr-oxygen cluster, resulting in the formation of oxygen-centered active sites within the cluster and the generation of porphyrin π-cation radicals within the ligands. This confirmed the ligand-to-cluster charge transfer (LCCT) process in MOFs (Figure 16c). In another study, EPR spectroscopy was used to elucidate the electron traps and transfer kinetics.^182^ The multiple signals at lower magnetic fields due to unpaired electrons in the π-conjugated aromatic ring of the macrocyclic metal ligand were observed at *g* = 2.0058, providing valuable insights for the production and subsequent transfer of a photogenerated charge carrier (Figure 16d). Combining EPR results with DFT calculations indicated that photoexcited electrons were predominantly localized on the aromatic branch of the porphyrin (Figure 16e, f). The active center and reaction intermediates of TiO_2_@PCN-222 photocatalytic CO_2_ reduction was further identified by EPR combined with in situ FTIR.^183^ As shown in Figure 16g, both pure PCN-222 and TiO_2_@PCN-222 exhibited distinct absorption peaks at *g* = 2.002, attributed to the oxygen vacancies of Zr–O clusters in Zr-MOF. In addition, the EPR signal peak of TiO_2_@PCN-222 was significantly enhanced under visible light irradiation, indicating that Zr–O clusters were the key active sites for photocatalysis. The emergence of additional weak peaks for TiO_2_@PCN-222 under light irradiation can be attributed to the Ti^3+^. In summary, Ti and Zr were the main active centers for the photocatalytic CO_2_ reduction of TiO_2_@PCN-222. Furthermore, the active intermediates, such as CO_2_^•‑^, m-CO_3_^2–^ and HCOO^–^, in the photocatalytic process were monitored by in situ FTIR, thereby elucidating the photoreduction mechanism of TiO_2_@PCN-222.

**Figure 16:**
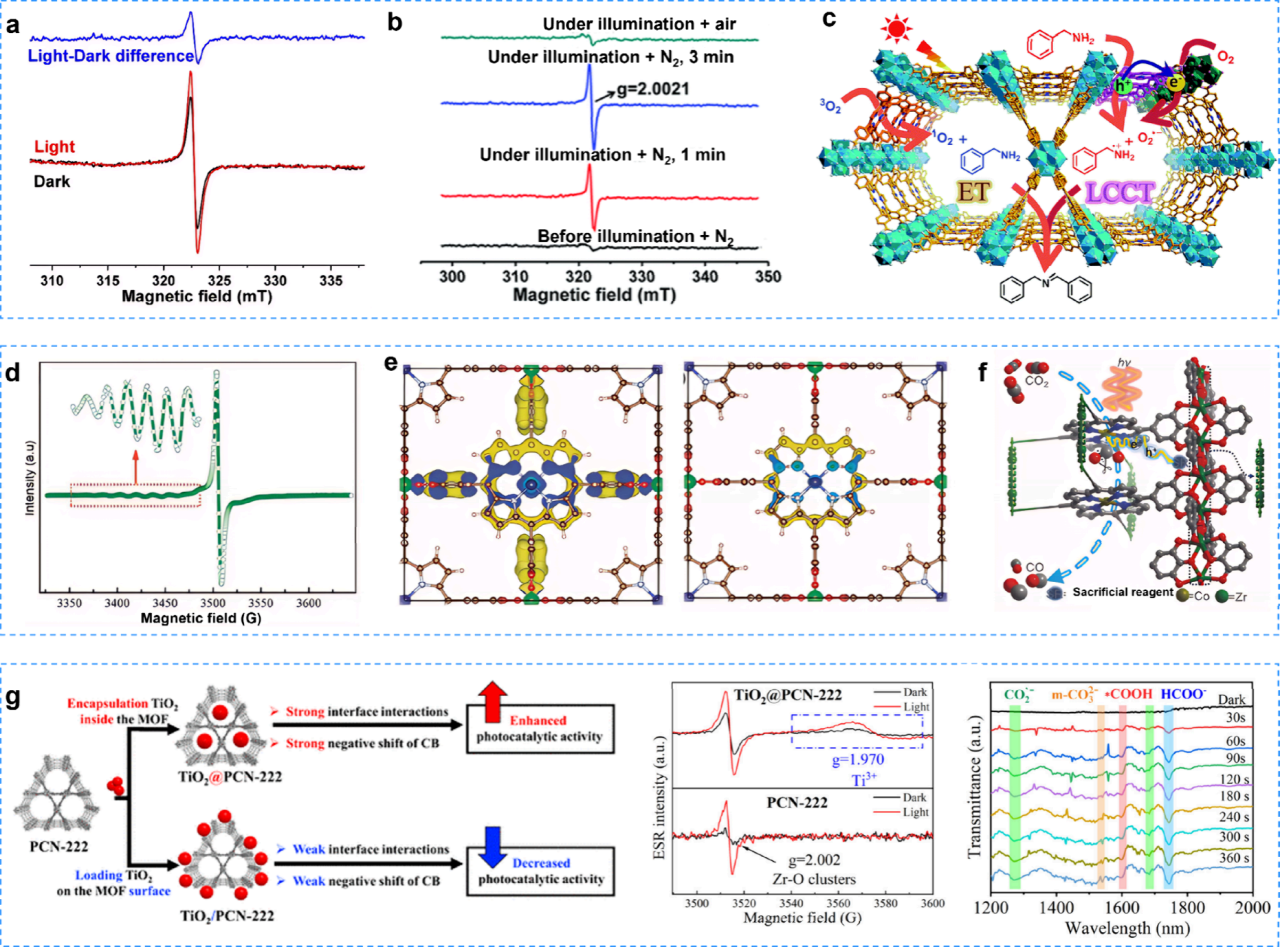
**a** and **b** EPR detection of PCN-222 under dark and light; **c** shows the charge transfer progress in PCN-222. Reproduced with permission from ref 181. Available under a CC-BY 3.0 license. Copyright 2018, The Authors, published by Royal Society of Chemistry. **d** The EPR spectrum; **e** partial charge density map; **f** photocatalytic mechanism for the CO_2_ reduction of ZrPP-1-Co. Reproduced with permission from ref 182. Copyright 2018, Wiley-VCH. **g** The synthetic strategy and EPR, FTIR signals for CO_2_ reduction on TiO_2_@PCN-222. Reproduced with permission from ref 183. Copyright 2024, American Chemical Society.

Despite its widespread application in catalysis research, EPR spectroscopy still possesses inherent limitation.^184^ Primarily, its time resolution is constrained to approximately 10 ns, thereby restricting its ability to detect specific intermediates. To address this issue, the use of spin trap reagents and the conduct of low-temperature experiments may be helpful. Moreover, achieving accurate quantification in EPR spectroscopy remains challenging due to the complex interaction of various factors including spin states, relaxation times, and experimental parameters, all of which profoundly influence signal intensity.^185^

## 5 Summary and Perspectives

This Review systematically introduces the semiconductor-like behavior of zirconium-based MOFs, elucidates the mechanisms governing their photocatalytic CO_2_ reduction, and summarizes strategies aimed at enhancing their photocatalytic performance. To gain insights into the underlying photocatalytic mechanism, various analytical techniques, including time- and space-resolved techniques such as TAS, XAS, DRIFTS and EPR, are discussed for tracking reaction intermediates. Although recent studies have highlighted the great potential of Zr-MOFs in photocatalytic CO_2_ reduction, it is acknowledged that challenges still exist, necessitating further efforts to facilitate advancements in this field.

Based on the aforementioned mechanisms of photocatalytic CO_2_ reduction reactions, the photocatalytic ability can be modulated through various chemical and physical properties of Zr-MOFs. These include the range of light absorption and associated bandgap values, the separation and transfer of photogenerated electrons and holes, the reduction/oxidation potentials, the structural integrity of pores and cavities, and the adaptable nature of pore sizes and volumes. Despite the promising advances made with these strategies, the overall performance of Zr-MOFs in photocatalytic CO_2_ reduction remains relatively modest, predominantly yielding C_1_ compounds like CO, HCOOH, CH_4_, and CH_3_OH, with fewer studies focusing on C_2_ reduction products. The integration of Zr-MOFs with inorganic semiconductors to construct composite materials or heterojunctions emerges as a potentially effective approach to enhancing C–C coupling and achieving the photoreduction of CO_2_ to C_2_ products. Here, we offer insights into the potential future development in the field of Zr-MOF photocatalysis.

1) The long-term stability of catalysts remains a critical issue in MOF photocatalysis. Although Zr-MOF has a strong skeleton stability compared with other metal MOFs, many Zr-MOFs still have long-term stability problems under harsh conditions such as strong acids and bases. An effective strategy to enhance the stability of Zr-MOFs is to utilize the hard and soft acid–base principle by employing bimetallic ligands that coordinate with metal ions. Alternatively, postsynthetic modification to introduce functional groups can improve the network rigidity and connectivity, further enhancing the stability of Zr-MOFs.
2) Zr-MOFs exhibit notable characteristics such as high porosity, specific surface area, and stability, yet the precise functions of channel types, cavities/pore sizes in the catalytic process remain underexplored, particularly for reactions involving selectivity and stereoisomerism. Moreover, the effect of intricate host–guest interactions between adsorbed molecules and the Zr-MOF framework on photocatalytic CO_2_ reduction has garnered limited attention. This potentially hold significant importance in substrate activation, enhancing photoinduced electron transfer, and potentially even lowering the redox potentials necessary for specific reactions.
3) The utilization of IR light in conventional semiconductors for photocatalysis presents a significant challenge, as IR light accounts for approximately 50% of the solar spectrum. Zr-MOFs with tunable organic ligands are promising candidates for photocatalytic reactions using IR light. Recently, Zeng and his colleagues designed a diverse series of porphyrin-based MOFs with unique π-electron configurations for CO_2_ photoreduction. The photoresponse capabilities were tunable spanning from visible light to IR wavelengths. Under IR light irradiation, the optimal MOF exhibited notable efficiency in reducing CO_2_ to HCOOH, achieving an impressive AQE of 1.11%.^186^
4) Achieving the desired overall reaction in photocatalysis without relying on sacrificial agents remains the ultimate goal. However, the sluggishness of the water oxidation reaction presents a formidable obstacle that must be overcome. Recent studies have highlighted bifunctional photocatalysis as a potential solution, integrating processes such as dye degradation or organic oxidation into the production of valuable chemicals with photocatalytic H_2_ production and CO_2_ reduction. This approach offers a sustainable alternative to conventional sacrificial agent consumption.^187^
5) Currently, photocatalytic CO_2_ reduction experiments are primarily conducted in pure and high concentration CO_2_ environments. Nevertheless, the CO_2_ emissions from industrial processes typically occur at low concentrations and are contaminated with O_2_, which swiftly quenches the reduction reaction. The Zr-MOFs have demonstrated great potential for achieving photoreduction of low-concentration CO_2_ due to their abundant porous structures that can effectively concentrate CO_2_, but relevant research is still in its infancy.^188^ It is expected that further progress will be made in achieving photocatalytic CO_2_ reduction under oxidizing conditions.
6) In addition to enhancing C–C coupling and achieve the photoreduction of CO_2_ to C_2_ products, new coupling strategies should be encouraged to be developed to obtain high-value products, such as implementing C–N, C–S and C–O coupling, etc. Besides, more advanced characterization techniques, such as time-resolved light emission electron microscope, transient surface photovoltage spectroscopy, surface photovoltage microscope, *in situ* Raman, *in situ* XPS, and *in situ* XAS, etc., should also be used to further investigate reaction mechanisms, including charge transfer kinetics behavior, reaction intermediates, and so on.

In conclusion, despite extensive research in photocatalysis over the past half-century, the exploration of Zr-MOFs for photocatalytic CO_2_ reduction remains in its nascent stage. Undoubtedly, Zr-MOFs possess significant advantages for photocatalytic CO_2_ reduction, including high surface area, high stability, compatibility, and precisely defined and tunable structural properties. We believe that Zr-MOFs will not only enhance our understanding of photocatalysis but also pave the way for the development of highly efficient catalysts for photocatalytic CO_2_ reduction.
